# Catabolism and interactions of syntrophic propionate- and acetate oxidizing microorganisms under mesophilic, high-ammonia conditions

**DOI:** 10.3389/fmicb.2024.1389257

**Published:** 2024-06-05

**Authors:** Nils Weng, Abhijeet Singh, Jonas A. Ohlsson, Jan Dolfing, Maria Westerholm

**Affiliations:** ^1^Department of Molecular Sciences, Swedish University of Agricultural Sciences, Uppsala, Sweden; ^2^Palaeobiology, Department of Earth Sciences, Uppsala University, Uppsala, Sweden; ^3^Faculty of Energy and Environment, Northumbria University, Newcastle upon Tyne, United Kingdom

**Keywords:** syntrophic propionate oxidation, syntrophic acetate oxidation, hydrogenotrophic methanogenesis, ammonia tolerance, anaerobic degradation, biogas production, syntrophy

## Abstract

Microbial inhibition by high ammonia concentrations is a recurring problem that significantly restricts methane formation from intermediate acids, i.e., propionate and acetate, during anaerobic digestion of protein-rich waste material. Studying the syntrophic communities that perform acid conversion is challenging, due to their relatively low abundance within the microbial communities typically found in biogas processes and disruption of their cooperative behavior in pure cultures. To overcome these limitations, this study examined growth parameters and microbial community dynamics of highly enriched mesophilic and ammonia-tolerant syntrophic propionate and acetate-oxidizing communities and analyzed their metabolic activity and cooperative behavior using metagenomic and metatranscriptomic approaches. Cultivation in batch set-up demonstrated biphasic utilization of propionate, wherein acetate accumulated and underwent oxidation before complete degradation of propionate. Three key species for syntrophic acid degradation were inferred from genomic sequence information and gene expression: a syntrophic propionate-oxidizing bacterium (SPOB) “*Candidatus* Syntrophopropionicum ammoniitolerans”, a syntrophic acetate-oxidizing bacterium (SAOB) *Syntrophaceticus schinkii* and a novel hydrogenotrophic methanogen, for which we propose the provisional name “*Candidatus* Methanoculleus ammoniitolerans”. The results revealed consistent transcriptional profiles of the SAOB and the methanogen both during propionate and acetate oxidation, regardless of the presence of an active propionate oxidizer. Gene expression indicated versatile capabilities of the two syntrophic bacteria, utilizing both molecular hydrogen and formate as an outlet for reducing equivalents formed during acid oxidation, while conserving energy through build-up of sodium/proton motive force. The methanogen used hydrogen and formate as electron sources. Furthermore, results of the present study provided a framework for future research into ammonia tolerance, mobility, aggregate formation and interspecies cooperation.

## Introduction

Anaerobic digestion (AD) is a waste management and renewable energy production technology which provides environmental benefits through substitution of fossil fuels and generation of nutrient-rich residual material that can be used as fertilizer. The AD sector has considerable expansion potential and can be one of the tools needed to prevent further climate change (World Biogas Association, 2019; EurObserv’ER, 2022). In AD, organic matter is degraded through a series of consecutive and parallel reactions by a consortium of cooperating microorganisms, eventually resulting in production of methane and carbon dioxide (CO_2_). Substrate composition and operating parameters have crucial impacts on the structure and activity of the microbial community, and thereby on overall process performance (Westerholm et al., 2019b). One of the most common process challenges is high ammonia levels arising from anaerobic degradation of protein-rich materials (e.g., slaughterhouse waste, food waste and animal manure) (Westerholm et al., 2022). High ammonia levels inhibit various members of the AD community, including acetate-utilizing methanogens (De Vrieze et al., 2015; Fischer et al., 2019; Wang et al., 2022), leading to lower methane yields and accumulation of the organic intermediates propionate and acetate (Westerholm et al., 2015). This acid accumulation leads to process instability and can halt upstream microbial activities, ultimately causing process failure if not managed properly.

Satisfactory methane yields and relatively stable operating conditions can still be achieved under high-ammonia conditions. In AD processes stressed by high ammonia or high temperature, syntrophic acid-oxidizing reactions have been shown to offer important routes for propionate and acetate conversion (Westerholm et al., 2016; Ruiz-Sánchez et al., 2018; Buhlmann et al., 2019; Singh et al., 2023). Within those routes, acetate is oxidized by syntrophic acetate-oxidizing bacteria (SAOB) that operate the Wood-Ljungdahl pathway (WLP) in reverse to form CO_2_ and hydrogen (H_2_) (or formate) (Schnürer et al., 1997; Hattori et al., 2005; Manzoor et al., 2016a). Propionate is metabolized by syntrophic propionate-oxidizing bacteria (SPOB), most often via the methylmalonyl-CoA (mmc) pathway which yields acetate, H_2_ and CO_2_ as products. This propionate conversion route occurs both in low- and high-ammonia conditions, although the bacterial species involved differ (Westerholm et al., 2022; Singh et al., 2023). A characteristic in common to both SAOB and SPOB is the need to establish tight mutualistic interaction with a hydrogenotrophic methanogen, which makes the acid oxidation reactions thermodynamically feasible by maintaining H_2_/formate at low levels. These mutualistic interactions and the thermodynamic restrictions underpinning them have been suggested to restrict acid degradation rates (Dolfing, 1992; Ishii et al., 2005; McInerney et al., 2009; Leng et al., 2018). Ammonia concentrations above certain levels can also affect cell growth and acid degradation rates despite the communities being ammonia-tolerant, ultimately leading to acid accumulation in AD processes (Westerholm et al., 2019a). To prevent acid accumulation and improve process disturbances, there is a need to increase understanding of the complex interactions that drive syntrophic acid oxidation in high-ammonia conditions, and the underlying principles. To date, transcriptomic activities of syntrophic propionate and acetate conversion to methane have only been studied in thermophilic enrichment cultures (Singh et al., 2023), while the growth characteristics and gene expression involved in syntrophic relationships between mesophilic acid oxidizers and their methanogenic partners remain underexplored.

The aim of this study was therefore to investigate mesophilic enrichment cultures of both acetate- and propionate-degrading communities during batch cultivation in high-ammonia conditions. Previous studies by our research group have shown that the mesophilic SAOB and SPOB co-exist in propionate-degrading communities under high-ammonia conditions (Singh et al., 2021, 2023). This raises the question of whether syntrophic acetate- and propionate-oxidizing bacteria employ similar strategies to cooperate with their hydrogenotrophic partner, and whether the behavior of SAOB is affected by the presence of SPOB. To study the interplay and growth of the syntrophic enrichment cultures, substrate consumption, product formation and pH during growth were monitored and thermodynamic constraints were calculated. Microbial composition over time in the cultures was explored using 16S rRNA gene profiling and metagenomics. Microbial activity was analyzed using metatranscriptomics, in order to reveal mutual and species-specific mechanisms that underpin the syntrophic cooperation.

## Materials and methods

### Source of microbial community

The enrichment cultures used originated from laboratory-scale continuous stirred-tank reactors (CSTRs) described previously (Singh et al., 2021). In brief, the CSTRs were inoculated with sludge from a biogas reactor degrading food waste supplemented with albumin and the resulting enrichment cultures were continuously fed with bicarbonate-buffered basal medium supplemented with ammonium chloride (0.3 M) and either 0.1 M sodium propionate or 0.1 M sodium acetate as substrate (Westerholm et al., 2015). The CSTRs were operated for more than 144 days before withdrawal of enrichment culture.

### Batch cultivation

**Figure 1:**
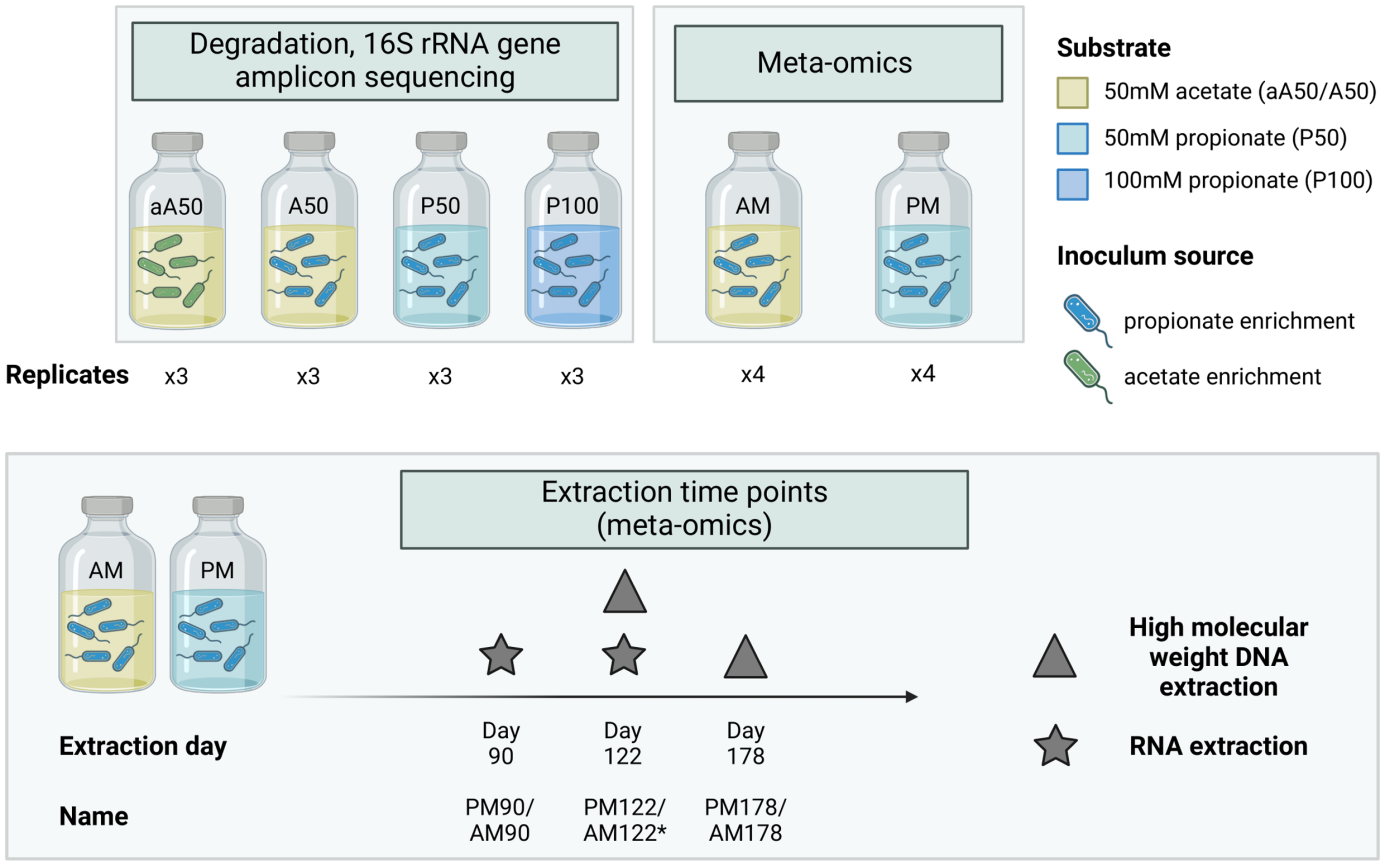
Experimental set-up used in batch cultivations. Triplicate cultures were prepared for assessment of acid conversion rates, 16S rRNA gene amplicon-based microbial community composition and thermodynamic calculations. Quadruplicate cultures were prepared for whole genome sequencing (WGS) and gene expression analysis, with an initial substrate concentration of either 50 mM acetate (AM) or 50 mM propionate (PM). A and P in batch names signify the provision of acetate or propionate as feedstock, respectively whereas 50 and 100 indicate the initial concentration in mM. The lowercase a in aA50 indicates inoculation with an enrichment culture originating from acetate-fed CSTR acetate, whereas other cultures were inoculated with an enrichment culture originating from a propionate-fed CSTR. *No RNA extraction conducted at day 122 in the acetate culture (AM), only DNA extraction.

Bicarbonate-buffered basal medium was prepared as described elsewhere (Westerholm et al., 2010) and supplemented with yeast extract (0.2 g/L) and ammonium chloride (0.3 M NH_4_Cl). The medium was then supplied with substrate, comprising either acetate (A) to an initial concentration of 50 mM (aA50, A50, AM) or propionate (P) to an initial concentration of 50 or 100 mM (P50, P100, PM) (Figure 1). Cultivation batches were prepared in serum bottles (1 L) sealed with butyl rubber stoppers. Each bottle contained a medium volume of either 0.5 L (degradation dynamics cultures) or 0.7 L (meta-omics cultures, PM, AM) and was autoclaved at 121°C for 20 min. After autoclaving, vitamins and trace elements were added, together with the reducing agents L-cysteine HCl (0.5 g/L) and Na_2_S (0.24 g/L) to remove traces of oxygen. All batches except aA50 were inoculated with 25–35 mL (5% of culture volume) of enrichment culture originating from the propionate-fed CSTR described above, while aA50 was inoculated with enrichment culture originating from the acetate-fed CSTR (denoted by lowercase “a” in batch name) (Figure 1). The pH of the medium after inoculation was 7.1–7.3. Triplicate samples were prepared for degradation dynamics cultures (aA50, A50, P50, P100) and quadruple samples for meta-omics cultures (AM, PM). Incubation proceeded under stationary conditions at 37°C in darkness.

To prevent accidental bursting of butyl rubber stoppers from the serum bottles due to overpressure, when the head-space gas pressure reached >1,000 mbar it was reduced to around 100 mbar above atmospheric pressure. The amount of methane released was accounted for by measuring gas composition and pressure before and after gas release.

In the batch assays used for meta-omics analysis VFA was sampled at day 0, 13, 62, 70 and 90 for the acetate fed culture (AM) and at day 0, 13, 62, 70, 90, 107 and 122 for the propionate fed culture (PM) (Supplementary Figure S1) The frequency of sampling was kept low to minimize disruption of syntrophic growth. At day 70, additional substrate was added to increase cell biomass for subsequent RNA extraction.

### Chemical analytical methods

For chemical and molecular analyses, weekly samples of gas (1 mL) and liquid (4 mL) were extracted from the batch assays employing syringes. Of the 4 mL liquid sample, 2 mL were allocated for pH measurements, while the remaining half was promptly frozen for subsequent utilization in VFA and molecular analyses. The pH was measured directly after sampling, using a pH electrode with an integrated temperature probe (InLab Expert Pro-ISM, Mettler Toledo, Ohio, United States). To quantify the concentrations of short-chain volatile fatty acid (VFA, acetate, propionate, butyrate, isobutyrate, valerate, isovalerate, caproate, isocaproate) the frozen 2 mL liquid sample underwent thawing and centrifugation (at 9000 *g*). The supernatant was subsequently injected into a high-performance liquid chromatography (HPLC) (Westerholm et al., 2010). Methane and CO_2_ levels in the headspace of the culture bottles were measured by gas chromatography as described previously (Westerholm et al., 2012). The H_2_ concentration in the headspace was measured using a gas chromatograph equipped with a reducing compound photometer detector (Peak Performer 1 Reduced Gas Analyzer PP1, Peak Laboratories, CA, USA) as described previously (Westerholm et al., 2019a). Pressure measurements of the headspace were conducted each time gas samples were taken using a handheld pressure meter (GMH 3111, Gresinger). For thermodynamic calculations, gas concentration measurements (CO_2_, CH_4_, H_2_) were converted to partial pressure by multiplication of the total headspace pressure. Total ammonia nitrogen (g NH_3_-N/L) was calculated for batch assays (aA50, A50, P50, P100) as a function of temperate and pH, as described by Hansen et al. (1998). The assumption was made that all of the initially supplemented ammonia (0.3 M) remained in the ammonia/ammonium form and was not converted to other compounds.

Methanogenic cell morphology was examined and micrographs were taken using a fluorescence microscope (Lumascope LS720 (Etaluma) at 60x magnification) and F420 autofluorescence of methanogens was visualized with a 370–410 nm excitation filter and a 429–462 nm emission filter.

### Thermodynamic calculations

The following equations were used in thermodynamic calculations:

Acetate oxidation to carbon dioxide and hydrogen:


(1) $$\begin{array}{l} CH_{3}COO^{-}+H^{+}+2H_{2}O\to 2CO_{2}+\\ 4H_{2}\Delta G{{}^{\circ}}^{\prime }=+54.9\mathrm{kJ}/\mathrm{mol} \end{array}$$

Propionate oxidation to acetate, carbon dioxide and hydrogen:


(2) $$\begin{array}{l} CH_{3}CH_{2}COO^{-}+2H_{2}O\to CH_{3}COO^{-}+\\ CO_{2}+3H_{2}\Delta G^{{}^{\circ}\prime }=+73.7\mathrm{kJ}/\mathrm{mol} \end{array}$$

Hydrogenotrophic methanogenesis:


(3) $$4H_{2}+CO_{2}\to CH_{4}+2H_{2}O\;\left(\Delta G^{{}^{\circ}\prime }=-130.8\mathrm{kJ}/\mathrm{mol}\right)$$

Values for the standard free energy of formation (G_f_) and the standard enthalpy of formation (H_f_) used for determining standard free energy change (ΔG_0_) for the reactions were based on values from Shock and Helgeson (Shock and Helgeson, 1990) for acetate/propionate and Hanselmann (Hanselmann, 1991) for the remaining species (H_2_, H^+^, CO_2_, H_2_O, CH_4_, Supplementary Data SE1). Free energy values for the reactions under nonstandard conditions, using the weekly VFA, gas and pH measurements were calculated as described elsewhere (Dolfing, 2016). The Gibbs-Helmholtz equation was applied to determine free energy at 310.15 K (37°C) in otherwise standard conditions:


(4) $$\begin{array}{l} \Delta G{{}^{\circ}}_{T_{310.15K}}=\Delta G{{}^{\circ}}_{T_{298.15K}}\times \left(\frac{T_{310.15K}}{T_{298.15K}}\right)+\\ \Delta H{{}^{\circ}}_{T_{298.15K}}\times \left(\frac{T_{298.15K}\times T_{310.15K}}{T_{298.15K}}\right) \end{array}$$

where *T* is temperature in Kelvin, ΔG°_T 298.15K_ is free energy of the reaction in standard conditions, ΔH°_T 298.15K_ is enthalpy of the reaction in standard conditions and ΔG°_T 310.15K_ is free energy of the reaction at 310.15 K (37°C) in otherwise standard conditions.

ΔG values were then calculated for the actual concentrations measured in cultures as:


(5) $$\Delta G=\Delta G{{}^{\circ}}_{T_{310.15K}}+\mathrm{RTlnQ}$$

where ΔG_0 T 310.15K_ is free energy in standard state conditions at 310.15 K (37°C), R is the gas constant, T is temperature in Kelvin and Q is the reaction quotient.

### 16S rRNA gene amplicon sequencing and data analyses

To investigate changes in the microbial community structure over time, the frozen 2 mL liquid samples taken weekly from the batch assays aA50, A50, P50, P100 and stored in −20°C were used for 16S rRNA gene amplicon sequencing. After thawing the samples were centrifuged (at 9000 *g*) and the pellets were used for sequencing and the supernatant was used for VFA analysis as described above. The DNA extraction was conducted using the FastDNA soil kit from MP Biomedicals (France) according to the manufacturer’s instructions. 16S rRNA gene amplicon libraries were constructed using primers 515F (GTGBCAGCMGCCGCGGTAA) and 805R (GACTACHVGGGTATCTAATCC) (Herlemann et al., 2011), and Illumina sequencing of the 16S rRNA gene amplicon libraries was performed as described by Müller et al. (2016). Paired-end sequencing was performed using Illumina MiSeq (Eurofins, Germany).

Raw paired-end reads were trimmed of primer sequences and Illumina adapters using Cutadapt (v 4.1) (Martin, 2011) and sequences with a maximum length of 300 bp were used in further processing. For generation of amplicon sequence variants, taxonomic assignment and abundance tables, the package dada2 (v 1.24.0) (Callahan et al., 2016) in R (v 4.2.1) (R Core Team, 2022) was used. In the DADA2 pipeline, forward and reverse sequences were cut to 159 and 191 bp, respectively, with a quality threshold of truncQ = 20, maxEE = (1,2), with cut lengths informed by calculations using the open-source software Figaro (Weinstein et al., 2019). For taxonomic assignment, a DADA2 formatted version of Genome taxonomy database (GTDB) release 207 was used as a reference rRNA database (Parks et al., 2018, 2020; Alishum, 2021). The R package phyloseq (v 1.40) (McMurdie and Holmes, 2013) was used for visualization of microbial community structure.

### DNA extraction and metagenomic assembly, binning, and functional analysis

For extraction of high molecular weight DNA suitable for long read sequencing, samples were taken toward the later stage of acid degradation at two occasions (day 122 and 178) for both meta-omic cultures (AM/PM, Figure 1). At day 122, 5 mL sample was sampled and pooled from each of the four biological replicates for both the AM or PM culture (total volume 20 mL for each setup) and at day 178, 20 mL sample was sampled from two out of the four biological replicates for both the AM and PM culture (total volume 40 mL from each setup). Samples were stored at −20°C until DNA extraction. Upon extraction, samples were thawed, centrifuged (at 4000 g, 4°C, for 20 min) and the supernatant removed, and pellets of biological replicates were pooled at this stage. DNA extraction was performed using NucleoBond^®^ AXG as described previously (Sun et al., 2020). The extracted DNA was barcoded using the ligation kit SQK-LSK109 together with the native barcoding expansion 1–12 (EXP-NBD104, Oxford Nanopore Technologies, Oxford, Great Britain) according to the manufacturer’s instructions. DNA concentration was determined using a 2,100 BioAnalyzer from Agilent. The extracted DNA was sequenced using a MinION device (Oxford Nanopore Technologies).

Raw Nanopore sequencing data were base-called and demultiplexed using guppy (v 5.0.7–1) (Wick et al., 2019) and filtered using filtlong (v 0.2.0) (Wick, 2022). Reads from the two different timepoints were merged and treated as a single acetate or propionate sample. Genome construction was performed using flye (v 2.8.3) (Kolmogorov et al., 2019) and subsequently polished using racon (v 1.4.20) (Vaser et al., 2017) and medaka (v 1.4.3) (Oxford Nanopore Technologies, 2024). Read mapping for polishing was performed with minmap2 (v 2.17) (Li, 2018). Binning was performed using three separate binning tools, Metabat2 (v 2.13) (Kang et al., 2019), concoct (v 1.1.0) (Alneberg et al., 2014) and Maxbin2 (v 2.2.7) (Wu et al., 2016). The bins generated from each tool were then consolidated to refined bins, using the bin_refinement module in metaWRAP (v 1.2.2) (Uritskiy et al., 2018), and the quality of the resulting bins was assessed using CheckM (v 1.0.18) (Parks et al., 2015). Bacterial metagenome assembled genome (MAG) annotations were conducted using Bakta (v 1.2.2) (Schwengers et al., 2021) and annotation of the archaeal MAG using Prokka (v 1.14.6) (Seemann, 2014). In addition, both archaeal and bacterial metagenomes were annotated, using GhostKoala (Kanehisa et al., 2016). Taxonomic assignments of MAGs were made using Sourmash (v 4.2.0) (Brown and Irber, 2016) against a reference database prepared from the Genome Taxonomy Database (GTDB, Release 202) (Parks et al., 2022).

In cases where MAGs from the acetate (AM) and propionate (PM) cultures were annotated as the same species, the highest-quality MAG was chosen for further studies. For higher-resolution annotation of hydrogenases, all coding sequences identified were classified against the curated hydrogenase database HydDB (Søndergaard et al., 2016) and true positives were identified by regular expressions searching for FeFe- or NiFe-binding motifs associated with hydrogenases (Vignais and Billoud, 2007; Calusinska et al., 2010) using Python (v 3.10.2). Regular expressions used for finding hydrogenase motifs are listed in Supplementary Data SE2. Any FeFe type A hydrogenases found residing in close genomic proximity (within 5 coding sequences) to a gene encoding NuoF were classified as bifurcating type A3 hydrogenases (Søndergaard et al., 2016). For comparison of the repertoire of hydrogenases for species closely related to the MAGs, protein FASTA files for those species were downloaded from RefSeq and annotated for hydrogenases as described above.

Prediction of signal peptides in all coding sequences was carried out using SignalP 6.0 (Teufel et al., 2022) and prediction of alpha and beta transmembrane proteins based on amino acid sequence using DeepTMHMM (v 1.0.24) (Hallgren et al., 2022). Species trees of MAGs were created using the species tree inference from all genes (STAG method) implemented in Orthofinder (v 2.5.4) (Emms and Kelly, 2017, 2019). For comparison of MAGs to their closest relatives, genome-to-genome distance was calculated using the Genome-to-Genome Distance Calculator (v 3.0) (Meier-Kolthoff et al., 2022) and whole genome average nucleotide identity (ANI) was determined using the python module PyAni (v0.2.9) (Pritchard et al., 2015), all with default parameters.

### RNA extraction, sequencing, and data analysis

For the acetate-containing cultures (AM, Figure 1), RNA was extracted at day 90 (AM90) toward the end of acetate degradation, when the acetate concentration was around 4 mM. For the propionate-containing cultures (PM), RNA was extracted on two occasions: on day 90 (PM90), when propionate was being degraded but was still present in high concentrations (40 mM), and on day 122 (PM122) when the propionate level was 11 mM. These specific sampling points were selected due to insufficient RNA yield during attempts at earlier stages of growth. RNA was extracted at day 122 (PM122) to ensure a time point with concurrent acetate and propionate degradation. Samples were taken from each of the four biological replicates and pooled prior to rRNA depletion, resulting in a total of three RNA samples being sequenced (AM90, PM90, PM122). To sample the flocs formed during cultivation, bottles were inverted prior to sampling to allow the flocs to sediment (approx. 5 min before sampling). Triplicate 50 mL culture broth samples were then collected from each bottle (including flocs), transferred to Falcon tubes pre-flushed with N_2_ and kept on ice. The Falcon tubes were immediately centrifuged at 4°C and 5,000 × *g* for 30 min. The supernatant was discarded and the triplicate cell pellets were pooled and dissolved in 1 mL chilled Trizol (TRIzol Reagent, Thermo Fisher Scientific, Massachusetts, United States) and 0.2 mL chloroform. Total RNA was then extracted using Quick-RNA Fecal/Soil Microbe Microprep Kit (Zymo Research, California, United States) with an additional DNase I depletion step. The biological replicates from RNA extraction were pooled, resulting in a total of three samples (AM90, PM90, and PM122). Ribosomal RNA was depleted using pan-prokaryote riboPOOL probes and streptavidin-coated Dynabeads (MyOne Streptavidin C1, Invitrogen #65001) according to the manufacturer’s protocol. The rRNA-depleted RNA was paired end sequenced (2× 75 bp) on a MiSeq v3 flow cell at the SNP&SEQ platform (SciLifeLab, Uppsala, Sweden).

RNA sequences were trimmed from adapters using Cutadapt (v 4.0) (Martin, 2011) and filtered for quality using Trimmomatic (v 0.39–2) (Bolger et al., 2014). Ribosomal RNA was filtered out *in silico* using SortMeRNA (v 2.1b) (Kopylova et al., 2012). Quality controlled and trimmed reads were then quantified by mapping against taxonomically distinct MAGs using Salmon (v 1.8.0) (Patro et al., 2017). Quantification results were reported as raw counts and normalized by the median of ratios method, using the R package DESeq2 (v 1.36.0) (Love et al., 2014). Further analyses and visualization of quantified expression were performed using the R packages pheatmap (Kolde, 2019) and ggplot2 (Wickham, 2016) and Python (v 3.10.2).

### Quantitative PCR

Quantitative PCR (qPCR) analyses were conducted in samples from the propionate enrichment batches P50 and P100. The qPCR was conducted using primers THACf (5´-ATCAACCCCATCTGTGCC-3′) and THACr (5´-CAGAATTCGCAGGATGTC-3′) (Westerholm et al., 2011) to quantify the 16S rRNA genes of *S. schinkii* and the primers MMBf (5´-ATCGRTACGGGTTGTGGG-3′) and MMBr (5´-CACCTAACGCRCATHGTTTAC-3′) (Yu et al., 2005) to determine the 16S rRNA gene level of methanogens of the order Methanomicrobiales. The qPCRs were performed in a 20 μL reaction mixture that consisted of 3 μL DNA sample, 10 μL iQ™ SYBR® Green Supermix (Bio-Rad), 1 μL of each primer (10 μM). The qPCR protocol for quantification was as follows: 7 min at 95°C, 40 cycles of 95°C for 40 s, annealing at 66 or 61°C (for the order Methanomicrobiales and *S. schinkii*, respectively) for 1 min and 72°C for 40 s, and melting curve analysis at 95°C for 15 s, followed by 1 min at 55°C and finally at 95°C for 1 s. All reactions were carried out in QuantStudio™ 5 (ThermoFisher).

## Results and discussion

### Biphasic propionate utilization and elevated relative abundance of syntrophic microorganisms

**Figure 2:**
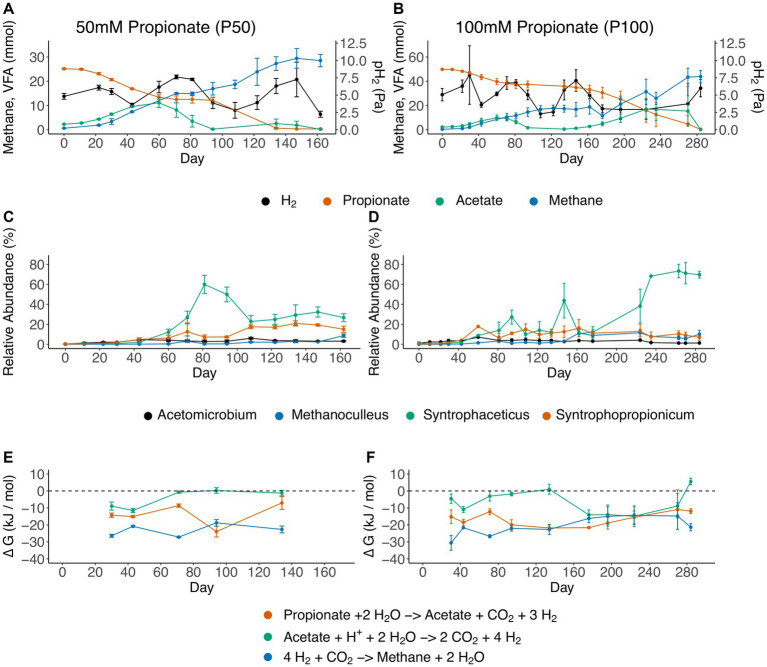
Overview of the propionate-degrading batch cultures, showing **(A,B)** substrate and product concentrations, **(C,D)** dynamics of genera that increased in relative abundance over the course of degradation and **(E,F)** change in Gibbs free energy (ΔG, kJ mol^−1^). Panels on the left refer to batches initiated with 50 mM propionate (P50), and panels on the right refer to batches initiated with 100 mM propionate (P100). Error bars indicate standard deviation of triplicates.

During all batch cultivations of the propionate enrichment culture, propionate degradation followed essentially the same pattern, irrespective of the starting concentration of propionate (50 or 100 mM). This pattern comprised a lag phase, initial propionate degradation with subsequent acetate accumulation, a plateau phase during which no propionate was degraded, and a second propionate degradation phase with concurrent acetate consumption (Figures 2A,B). For the 100 mM setup a second acetate peak was observed (17 mM at day 224) whereas minor acetate accumulation was observed for the 50 mM setup. This discrepancy could be explained by the longer plateau phase, in which the acetate oxidizing community is starved on substrate, as well as overall greater net conversion of propionate 100 mM setup. The findings from the 16S rRNA gene amplicon sequencing demonstrated the initial dominance of two members from the Tissierellaceae family (Supplementary Figure S2) within the Clostridia class (Supplementary Figure S3). Given the gradual decline in the relative abundance of Tissierellaceae over time, it’s plausible that these community members were not directly engaged in acid degradation. Instead, they likely converted compounds found in the yeast extract or the reducing agent cysteine.

Furthermore, the results showed that initiation of propionate degradation by day 22 in P50 and day 30 in P100 was associated with 2% increased relative abundance of “*Ca.* Syntrophopropionicum”. The maximum relative abundance of “*Ca.* Syntrophopropionicum” was 22% in P50 (day 177) and 18% in P100 (day 60) (Figures 2C,D; Supplementary Figure S2). During the initial propionate degradation phase, acetate accumulated stoichiometrically in approximately 1:1 ratio with propionate degradation (Figures 2A,B; Supplementary Data SE3), indicating that no acetate was consumed during the initial phase of propionate degradation. Subsequent cessation of propionate degradation and a decline in acetate levels (P50: day 81, P100: day 71) coincided with an increase in relative abundance of a bacterium of the genus *Syntrophaceticus* up to a maximum of 58 and 71% for P50 (day 81) and P100 (day 263), respectively (Figures 2A–D). The qPCR results revealed *S. schinkii* 16S rRNA gene abundance of 10^6^ copies/ng DNA during the first 80 days in P50 and P100. In accordance with the amplicon sequencing, a decrease in *S. schinkii* copy numbers (by a factor of ten) was observed as acetate levels declined (between days 94–134 in P50 and days 94–162 in P100, Figure 1; Supplementary Figure S4). However, as the experiment progressed, the counts gradually increased, ultimately reaching their initial levels. The methanogenic community initially accounted for less than 0.02% of total reads and consisted of a single species belonging to the genus *Methanoculleus.* The relative abundance increased over time, reaching a final value of 10–13% (Figures 2C,D). To compensate for restricted primer specificity of methanogens by the Illumina sequencing primers, qPCR analysis of the order Methanomicrobiales was conducted. The result demonstrated an average level of 10^8^ 16S rRNA gene copies/ng DNA during the initial experimental period (day 0 to 94/134). Similar to the SAOB, the gene copy numbers decreased tenfold between days 94–134/162 in P50 and P100, followed by a subsequent increase to initial levels (Supplementary Figure S4).

**Figure 3:**
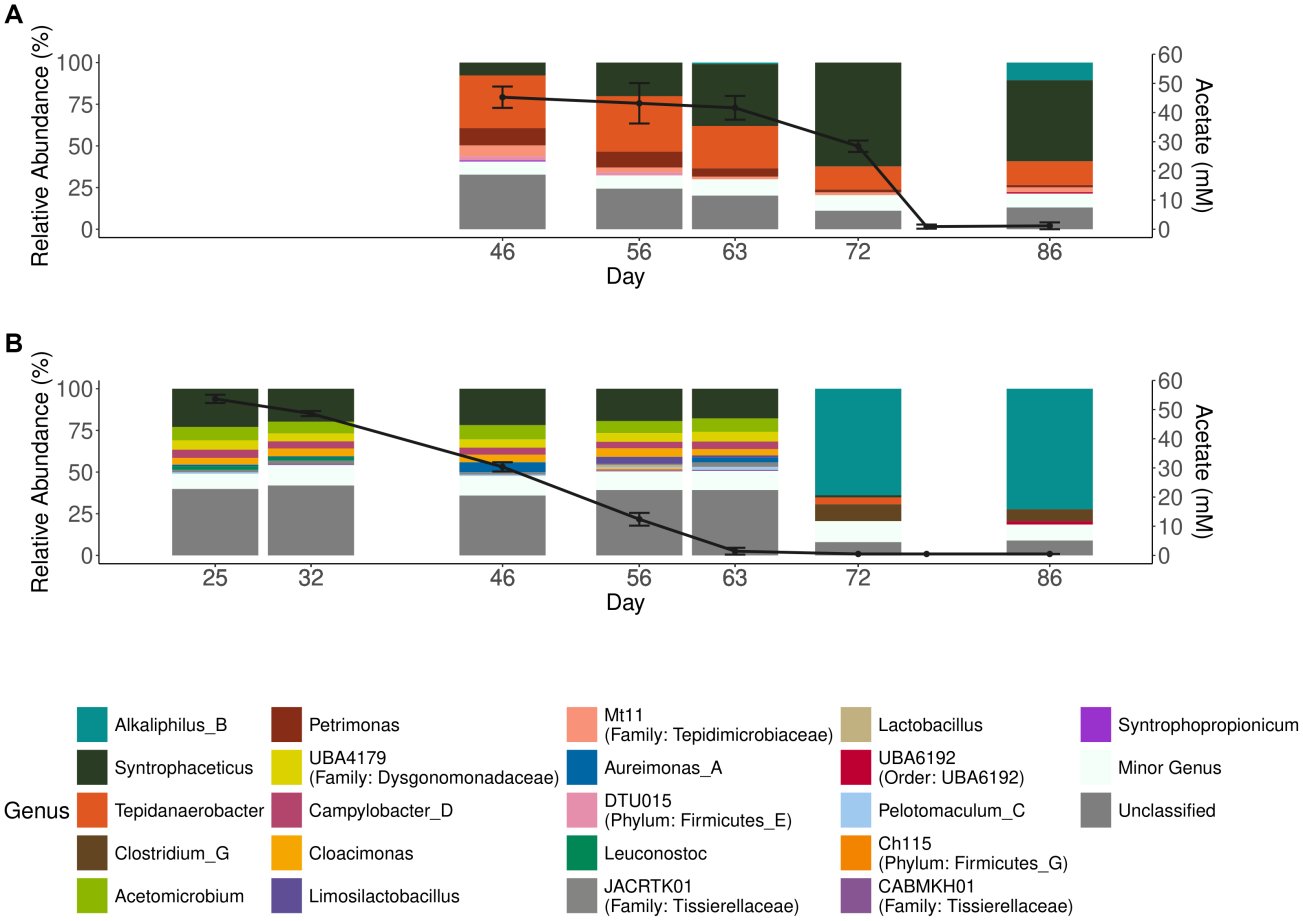
Acetate concentration and microbial community structure of the acetate-degrading batch cultures. Acetate concentration (black lines, right axis) and relative abundance (>2.5%) of microbial communities (bar plots, left axis) in batches inoculated with enrichment culture from **(A)** propionate-fed CSTR (A50) and **(B)** acetate-fed CSTR (aA50). Error bars indicate standard deviation of triplicate samples.

To investigate degradation of acetate by the microbial community enriched for either acetate (aA50) or simultaneous propionate and acetate degradation (A50), batch cultures with acetate as substrate were prepared and inoculated with either acetate (aA50) or propionate (A50) CSTR enrichment cultures (Figure 1). Similar to P50 (and P100 cultures), acetate degradation in A50 coincided with a rapid increase in the genus *Syntrophaceticus* (Figure 3A). In aA50, on the other hand, the genus *Syntrophaceticus* remained relatively constant over the course of degradation. After acetate depletion in aA50 (<0.2 g/L, day 72) (Figure 3), a drastic shift in community structure was observed and members of the genera *Alkaliphilus* and *Clostridium* increased to relative abundance of 70 and 10%, respectively. The cause of this change in the microbial community remains unknown and requires further investigation. A similar shift was not seen in the A50 community. No methanogenic amplicon sequence variants were identified in these cultures. Other bacteria present in all propionate- and acetate-fed batches over the course of degradation were members of the genera *Tepidanaerobacter* and *Acetomicrobium*, each representing relative abundance of 1–9% of the total community at the end of the trials (Figure 3, Supplementary Figure S5). Of the approximately 24% of the total community not classified at genus levels, a majority (73%) were affiliated to the Clostrida class (data not shown). For the acetate fed batches 20% lacked classification at genus level, and of the unclassified 52% were Clostridia, 12% Campylobacteria, 9% Bacterioidia. Notable difference between the acetate-fed batches was the presence of fewer different genera in the A50 culture than in the aA50 culture (Figure 3). This could relate to the long starvation time for the acetate-consuming populations during previous batch cultivations of A50 (i.e., before enough acetate is formed from the propionate degradation) or because propionate itself had an inhibitory effect on some of the species that thrive on yeast extract, cysteine or other compounds in the cultivation medium (Han et al., 2020).

### Thermodynamic calculations indicate low impact of acetate levels on propionate degradation

The free energy of VFA oxidation and methanogenesis under the non-standard conditions, calculated based on weekly measures of gas composition, VFA, pH and temperature, remained exergonic throughout the cultivation period (Figures 2E,F; Supplementary Data SE3). One exception was that acetate oxidation (equation 1) reached slightly positive ΔG values both during low and high propionate settings (+0.25 kJ/mol / +5.5 kJ/mol), when acetate levels were very low (at day 94 in P50 and day 134/280 in P100). The ΔG values for propionate oxidation (equation 2) fluctuated between −25 kJ/mol and − 10 kJ/mol during the experiment. The ΔG values for hydrogenotrophic methanogenesis (equation 3) fluctuated between −30 kJ/mol and − 10 kJ/mol throughout cultivation.

Even though propionate oxidation was exergonic (> −10 kJ/mol throughout cultivation) a halt in propionate degradation was observed coinciding with increasing acetate levels. Therefore, potential acetate inhibition was analyzed using thermodynamic calculations. The critical acetate concentration needed to reach thermodynamic equilibrium (ΔG = 0) for propionate degradation was calculated using fixed values for propionate (58 mM), pH_2_ (5.3 Pa) and pCO_2_ (26,700 Pa) (equations 4,5). These fixed values were based on the mean experimental conditions during the plateau phase of propionate degradation (P100, day 81–134). The critical acetate concentration needed for propionate oxidation to become endergonic was around 4.3 M, i.e., orders of magnitude higher than the actual acetate concentrations in the experimental set-up (Supplementary Data SE4). Therefore, from a thermodynamic point of view the halt in propionate degradation was not explained by increasing acetate level.

As the stoichiometric coefficient for H_2_ during syntrophic acetate and propionate oxidation is 4 and 3, respectively (equations 2, 3), the H_2_ partial pressure has a distinctly greater impact on the ∆G values of these reactions as compared to other reactants and products (as their coefficients are lower). Thus, it is critical that H_2_ concentrations are kept low by a syntrophic partner. To confirm that this is the case in the observations, the Pearson correlation of H_2_ partial pressure and acetate degradation rates (based on slope of linear model fitted through the particular day in question) was analyzed. Pearson correlation coefficient of −0.30 (*p* < 0.01) was found, indicating a significant negative impact of H_2_ partial pressure on acetate degradation rates, which was expected given the stoichiometry of acetate oxidation (equation 1).

Despite several similarities between the 50 mM and the 100 mM cultures in terms of community and degradation profiles, notable differences complicate drawing overarching conclusions. In the 50 mM setup, the cessation of propionate degradation could potentially be attributed to a biological interplay between methanogens and the two acid oxidizers. This speculation stems from the observation that the halt in propionate degradation and lowering of acetate levels coincide with an increase in *Syntrophaceticus* abundance, accompanied by a rise in hydrogen partial pressure (Figure 1). It is plausible that the additional hydrogen and/or formate (or other reducing equivalents) from an increased activity of the SAOB exceed the capacity of the existing methanogenic population, resulting in elevated hydrogen levels, which could have become detrimental to propionate degradation. Subsequently, once all acetate is consumed and hydrogen levels decline, propionate oxidation resumes without acetate accumulation. Although the transition shows similar dynamics, in the 100 mM setup propionate oxidation does not resume directly after acetate gets depleted. Instead it took 40 days before propionate degradation recommenced. The qPCR results also indicated that the gene abundance of Methanomicrobiales required somewhat longer levels to return to 10^8^ 16S rRNA gene copies/ng DNA after the intermediate decrease (Supplementary Figure S4), which further point to a biological interplay between methanogens and the two acid oxidizers. Labeling experiments and metaproteomics could potentially provide further information to reveal the underlying mechanisms giving rise to this turn-taking behavior.

### Description of the four transcriptionally active MAGs obtained from the syntrophic enrichment culture

**Figure 4:**
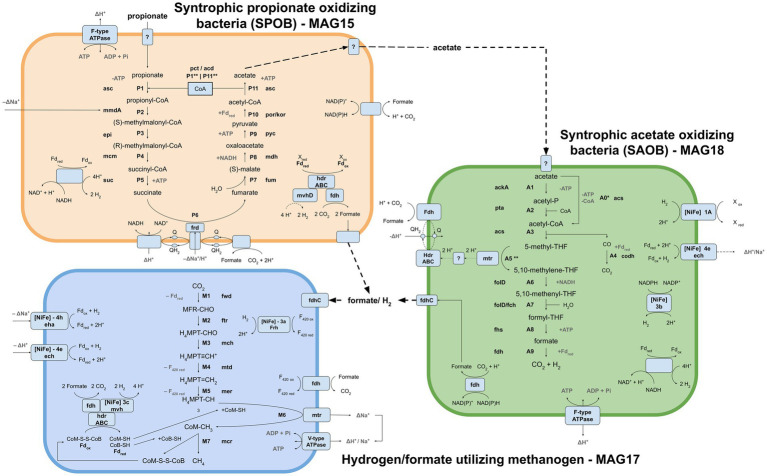
Visualization of the metabolic pathways and interplays employed for propionate oxidation by the syntrophic propionate-oxidizing bacteria (SPOB, MAG15) via acetate assimilation by the syntrophic acetate-oxidizing bacteria (SAOB, MAG18) and hydrogen/formate conversion to methane by the hydrogenotrophic methanogen (MAG17) under mesophilic and high-ammonia conditions. SPOB: asc acetyl-CoA synthetase, pct propionate CoA-transferase, acd acetate CoA ligase, mmdA methylmalonyl CoA decarboxylase, epi methylmalonyl CoA/ethylmalonyl CoA epimerase, mcm methylmalonyl-CoA mutase, suc succinyl-CoA synthetase, frd succinate dehydrogenase, fum fumarate hydratase, mdh malate dehydrogenase, pyc pyruvate carboxylase, por/kor pyruvate ferredoxin oxidoreductase/2-oxoglutarate/2-oxoacid ferredoxin oxidoreductase. SAOB: ackA acetate kinase, pta phosphate acetyltransferase, codh anaerobic carbon-monoxide dehydrogenase, fold methylenetetrahydrofolate dehydrogenase, fch methenyltetrahydrofolate cyclohydrolase, fhs formate-tetrahydrofolate ligase. Methanogen: fwd formylmethanofuran dehydrogenase, ftr formylmethanofuran-tetrahydromethanopterin N-formyltransferase, mch methenyltetrahydromethanopterin cyclohydrolase, mtd methylenetetrahydromethanopterin dehydrogenase, mer 5,10-methylenetetrahydromethanopterin reductase, mtr tetrahydromethanopterin S-methyltransferase, mcr methyl-coenzyme M reductase, eha energy-converting hydrogenase, ech ech hydrogenase, Frh NiFe type 3a hydrogenase. Generic: Fd ferredoxin, hdr heterodisulfide reductase, mvhD F_420_-non-reducing hydrogenase, fdh formate dehydrogenase, Q quinone, NuO NADH:ubiquinone oxidoreductase, fdhC formate transporter.

Metagenomic binning resulted in ten and nine MAGs (>72% completeness) from the acetate and propionate cultures, respectively. Five of the MAGs retrieved from the acetate- and propionate-fed batches had identical taxonomic assignments, while four MAGs lacked taxonomic assignment on superkingdom level (Supplementary Data SE5). In the metatranscriptomic data, around 41% of the reads were removed as rRNA. The majority of the remaining reads were mapped against MAG17 (61%), followed by MAG18 (23%), MAG16 (4.7%) and MAG15 (3.7%). Based on microbial community structure (Figure 2), the taxonomic placement of the MAGs (Supplementary Table S1) and metatranscriptomic mapping (Supplementary Figure S6), MAG15, MAG17 and MAG18 were identified as most important for the metabolic activities of the communities (Figure 4). Of the transcriptionally active metagenomes MAG16 and MAG17 were of high quality (completeness >90%, contamination <5%) while MAG15 and MAG18 were of medium quality (completeness >80%, contamination <10%) (Supplementary Table S2). A detailed description of the characteristics of these MAGs, their functional activities and pathways is provided below.

### The putative SPOB *Ca.* “Syntrophopropionicum ammoniitolerans” (MAG15)

**Figure 5:**
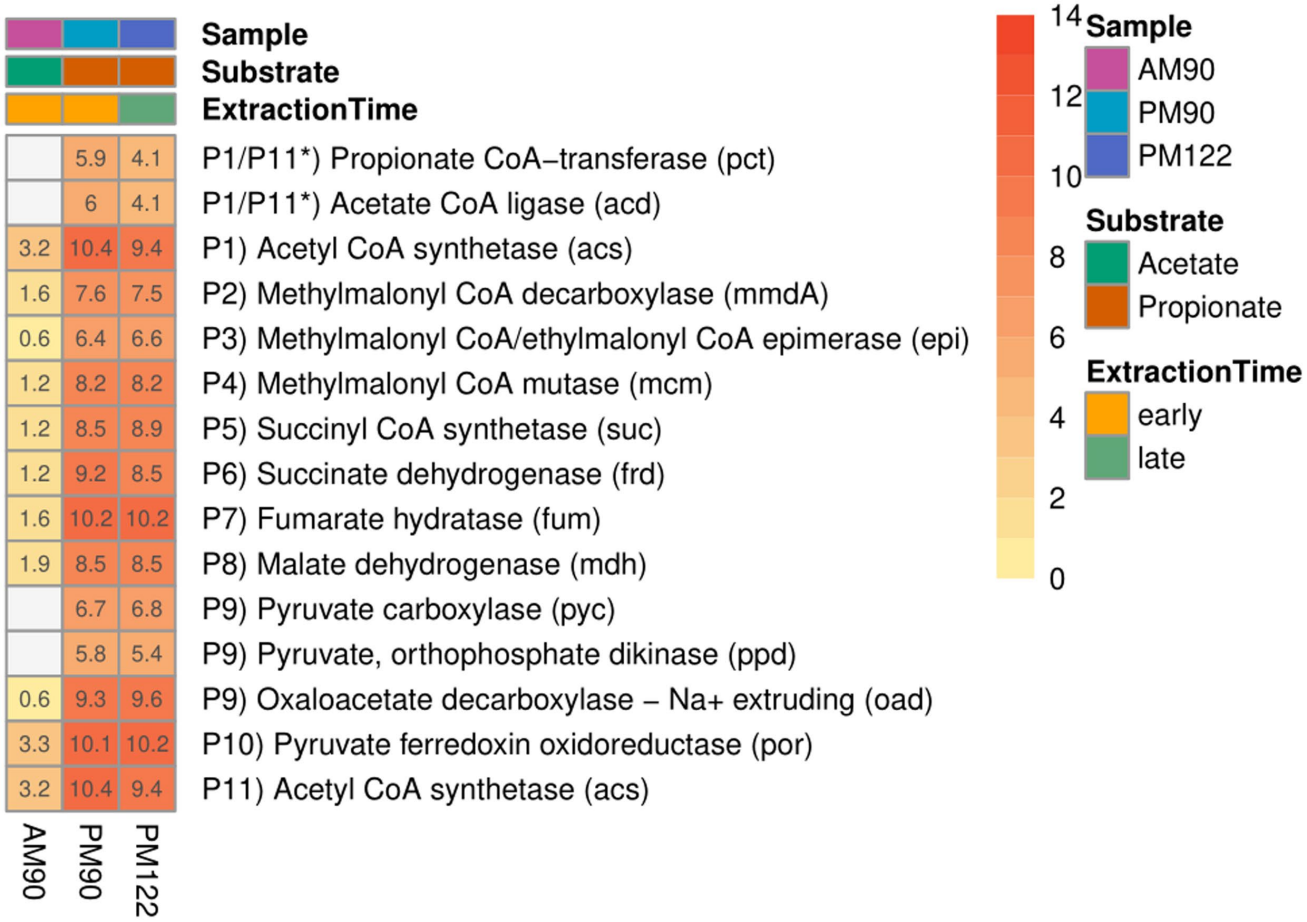
Expression of genes involved in the methylmalonyl-CoA pathway by the putative propionate oxidizer “*Ca.* S. ammoniitolerans” (MAG15) in propionate (PM90, PM122) and acetate batch assays (AM90). Heatmap values are aggregated log2-transformed deseq2 normalized count, of all copies and subunits for each respective gene. Genes not expressed are shown in gray. *Putative coupling between step P1 and P11 for propionate activation.

Taxonomic analysis identified MAG15 as the putative SPOB “*Candidatus* Syntrophopropionicum ammoniitolerans” (family *Pelotomaculaceae*, ANI 99.4%) (Supplementary Table S1; Supplementary Figure S7). This bacterium was previously characterized in an in-depth study of the high-ammonia reactors that supplied the inoculum used in the present study (Singh et al., 2021). The present study confirmed that genes for all steps of the conventional methylmalonyl-CoA pathway were expressed by MAG15 in the propionate cultures, whereas little or no expression was shown in the acetate culture (Figure 5; Supplementary Data SE6). These results strongly indicate that MAG15 is the main propionate oxidizer in the community representing the SPOB candidate “*Ca.* S. ammoniitolerans”.

As reported for all known SPOB in the family Pelotomaculaceae (Kato et al., 2009), most of the genes coding for enzymes in the methylmalonyl-CoA pathway formed an operon-like cluster in MAG15 (except the genes for steps P1, P6 and P11; see Figure 4 and Supplementary Data SE6). The transcriptomic data revealed that MAG15 exhibited relatively low expression of genes for CoA transferases in comparison with the highly expressed AMP-dependent acetyl-CoA synthetase (acs). This indicates that, as reported for several other SPOBs (Kato et al., 2009; Hidalgo-Ahumada et al., 2018; Hao et al., 2020; Singh et al., 2023), “*Ca.* S. ammoniitolerans” MAG15 connects the first step of endergonic propionate activation (P1) (Sofeo et al., 2019) with the last step of exergonic acetyl-CoA de-activation (P11). For the subsequent step involving endergonic addition of a carboxyl group to propionyl-CoA (P2), two catalytic paths have been proposed. One involves coupling of this step with the downstream exergonic decarboxylation of oxaloacetate (P9) catalyzed by methylmalonyl-CoA carboxyltransferase. In the other option, methylmalonyl-CoA decarboxylase (mmdA) driven by sodium import catalyzes the reaction, which is most likely the case for MAG15 based on its expression of mmdA, located in the aforementioned operon-like cluster (Supplementary Data SE6, Locus: BGEMGI_09260).

Expression of pyruvate carboxylase subunit B (pycB) and an adjacent gene annotated as oadA located in the MMC operon-like cluster indicated their involvement in step P9. In the SPOB candidate “*Ca.* Propionivorax syntrophicum”, conversion of oxaloacetate to pyruvate has been proposed to proceed through extrusion of two sodium ions by oxaloacetate decarboxylase (oad) (Hao et al., 2020). However, “*Ca.* S. ammoniitolerans” MAG15 only encoded and expressed the oadA subunit, and BlastP suggested similarities of this gene with pycB, suggesting that it did not employ sodium ion extrusion at this step and instead relied on the ATP forming pyruvate carboxylase. Detailed descriptions of gene expression by MAG15 for the remaining reaction steps in the MMC pathway are given in Supplementary Note 1.

### The SAOB *Syntrophaceticus schinkii* (MAG18)

**Figure 6:**
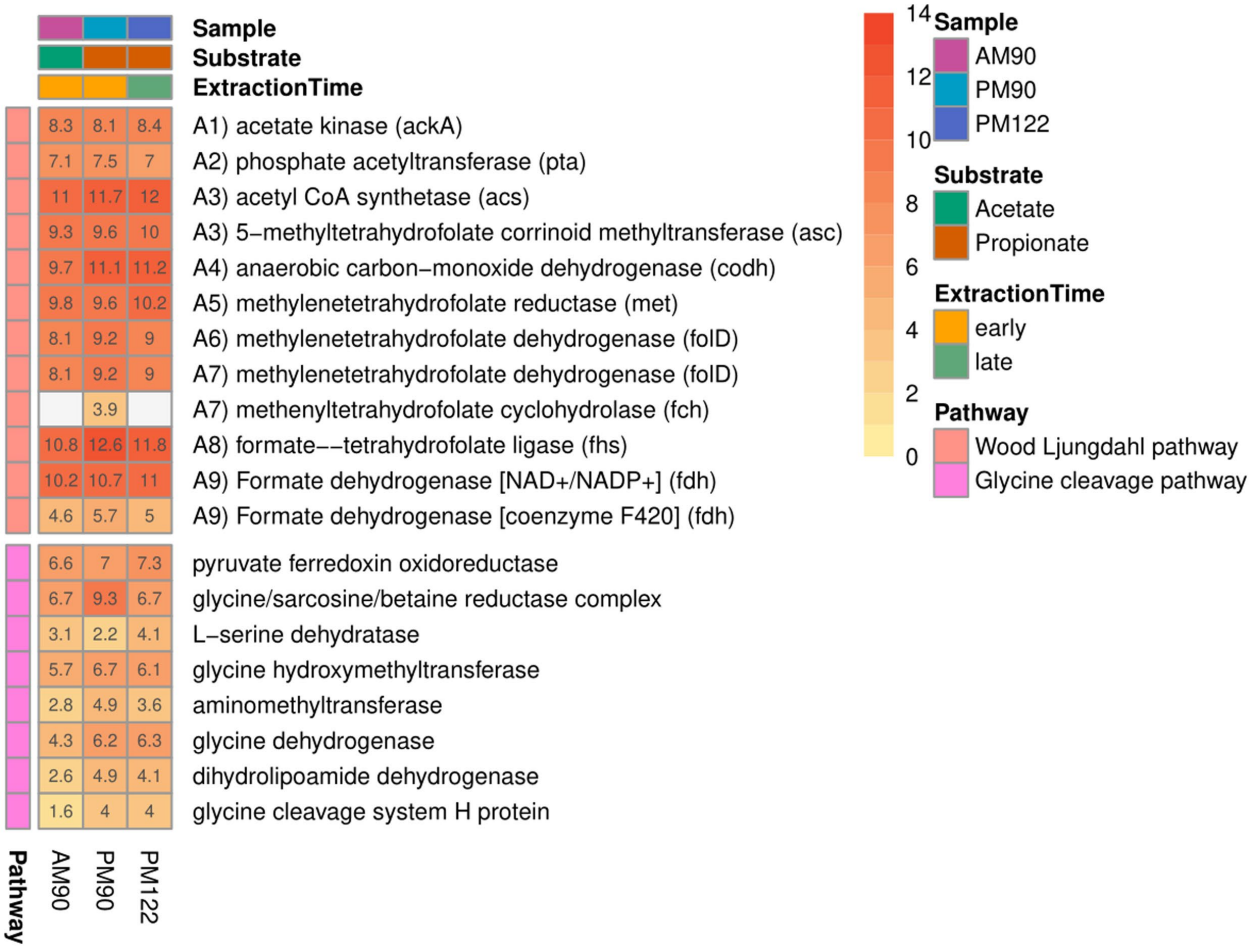
Expression of genes in the Wood-Ljungdahl pathway by the syntrophic acetate-oxidizing bacteria (SAOB) *S. schinkii* (MAG18), in propionate (PM90, PM122) and acetate batch assays (AM90). Heatmap values are the aggregated log2-transformed deseq2 normalized count, of all copies and subunits for each respective gene. Genes not expressed are shown in gray.

Genomic analysis of MAG18 indicated 98.6% ANI to the SAOB *Syntrophaceticus schinkii* (Supplementary Table S1), previously isolated from a high ammonia mesophilic biogas digester (Westerholm et al., 2010). The metatranscriptomic results showed that MAG18 had the second most abundant expression level (after the methanogen) in both the acetate-fed and propionate-fed batches (Supplementary Figure S6). These results demonstrate that MAG18 is the previously known SAOB *S. schinkii* and the main acetate oxidizer in both the AM and PM enrichment communities. The Wood-Ljungdahl Pathway (WLP) is believed to operate in the reverse direction during syntrophic acetate oxidation in the presence of a reducing equivalents-consuming partner. In MAG18, all genes involved in the WLP were expressed in both the acetate- and propionate-fed batches (Figure 6). The gene expression pattern found for MAG18 was in agreement with previous findings for *S. schinkii* (Schnürer et al., 1997; Hattori et al., 2005; Oehler et al., 2012; Manzoor et al., 2016a), showing initial endergonic activation of acetate to acetyl-CoA by an ATP-consuming acetate kinase (ack) followed by exchanging the phosphate group for a CoA group using phosphate acetyltransferase (pta) (Figures 4, 6 steps A1-A2). MAG18 also expressed acetyl-CoA synthetase, enabling direct activation of acetate to acetyl-CoA, although the gene expression was lower than that of *ack* and *pta*. The thermophilic SAOB *T. phaeum* has been suggested to activate acetate through an ATP-independent aldehyde ferredoxin oxidoreductase followed by oxidation of acetaldehyde to acetyl-CoA (Keller et al., 2019), which would allow the bacterium to balance the overall ATP budget. However, low transcript levels of the corresponding genes compared with that of acetate kinase in MAG18 (Supplementary Data SE6) indicated that ATP was consumed in this first step, as shown previously for a thermophilic ammonia-tolerant SAOB candidate (Singh et al., 2023).

For the carbonyl branch, MAG18 expressed genes for the ferredoxin-dependent carbon monoxide dehydrogenase (CODH) reducing carbon monoxide to carbon dioxide (Figures 4, 6; step A4). The CODH was encoded in close proximity to the methylenetetrahydrofolate reductase (MTHFR) and dehydrogenase required for the methyl branch of the WLP (steps, A5-A7; Figures 4, 6). MAG18 also expressed genes for heterodisulphide reductase-like protein (hdrABC), with subunit A located within the CODH operon and the methyl-branched genes and subunits B-C located roughly 30 coding sequences upstream of this operon (Supplementary Figure S8). Notably, many of the interposed genes coded for heme and ubiquinone synthesis, both typical electron carriers, and their expression by MAG18 could be an indication of their involvement in the methyl branch. A second hdrABC-containing gene cluster was located next to those for a formate dehydrogenase and a methyl viologen-reducing hydrogenase (mvhD). Similar gene organization has been reported for *T. phaeum* (Keller et al., 2019), with the exception that no genes for mvhD were located next to the formate dehydrogenase in *T. phaeum* (Supplementary Figure S8). In SAOB, the presence of the endergonic methyl-THF oxidation (step A5) remains an enigma, as it releases electrons with a redox potential of −200 mV, which cannot be directly transferred to NADH (redox potential of −320 mV) (Thauer et al., 1977). In *T. phaeum*, methyl-THF oxidation has been suggested to be linked with formate formation, through a (reversed) electron transport chain involving hdrABC, a quinone pool and membrane-bound formate dehydrogenase, where the endergonic threshold is surmounted by a proton motive force. For *T. phaeum*, such a proton gradient may be created through ATP hydrolysis, since the species was able to achieve acetate activation without ATP investment, thus netting 1 ATP for each acetate oxidized. As seen for a thermophilic and ammonia-tolerant SAOB candidate (Singh et al., 2023), this is not a viable option for MAG18 since ATP investment is needed for the initial acetate activation. However, both MAG18 and the ammonia-tolerant SAOB candidate express a gene for a membrane-bound hydrogenase complex (Ech) that could serve as a potential driver of a proton motive force through cytoplasmic proton consumption. However, irrespective of the source of the proton motive force, the expression profile of MAG18 and the thermophilic ammonia-tolerant SAOB candidate indicated that a mechanism involving hdrABC and formate dehydrogenase, similar to that observed for *T. phaeum*, is utilized for methyl-THF oxidation (step A5, Figures 4, 6). This reinforces the hypothesis put forward by Keller et al. (2019) that a periplasmatically orientated formate dehydrogenase could be key for reversibility of the WLP. However, further research is needed to confirm the metabolic activity underpinning the oxidation of methyl-THF (step A5) in *S. schinkii*, especially with respect to the establishment of a proton gradient.

### SPOB exclusively expressed [FeFe]-type hydrogenases, while the SAOB expressed both [FeFe] and [NiFe] hydrogenases for electron transfer

Syntrophs often express multiple variants of hydrogenases and formate dehydrogenases, which serve as an important outlet for re-oxidation of reduced electron carriers formed during VFA oxidation (Sieber et al., 2012). Genes encoding both formate dehydrogenases and hydrogenases were expressed by the SPOB candidate “*Ca.* S. ammoniitolerans” MAG15 and the SAOB *S. schinkii* MAG18, indicating that both use H_2_ and formate as outlets for excess reducing equivalents generated during acid oxidation. “*Ca.* S. ammoniitolerans” MAG15 only expressed genes for [FeFe]-type hydrogenases, which were all predicted to be cytoplasmic and included several bifurcating type A3 [FeFe] hydrogenases, one of which was in close genomic proximity to a sensing type C3 [FeFe] hydrogenase. Absence of [NiFe] hydrogenases is a shared characteristic of SPOB in the genus *Desulfofundulus*, whereas SPOB belonging to the genus *Pelotomaculum,* the thermophilic ammonia-tolerant SPOB candidate “*Ca.* T. ammoniitolerans” and *S. fumaroxidans* express both [FeFe] and [NiFe] hydrogenases (Westerholm et al., 2022; Singh et al., 2023).

For acetate oxidation, *S. schinkii* MAG18 expressed a gene for a bifurcating [FeFe]-type A3 hydrogenase. It also expressed genes for multiple [NiFe] hydrogenases, which included a periplasmic type 1a hydrogenase, a NADP-coupled type 3b hydrogenase and a membrane-bound hydrogenase complex (Ech) type 4e hydrogenase (Figure 4). Comparison of the thermophilic SAOB *T. phaeum* with the mesophilic *S. schinkii* MAG18 revealed a similar set of hydrogenases (NiFe 1a, 4e, 3b and FeFe A3), except that *T. phaeum* also had a membrane-bound NiFe 4a formate hydrogenlyase (Supplementary Figure S9).

At this point, it is difficult to draw conclusions from the observed differences (i.e., MAG15 only expressed [FeFe]-type hydrogenases, MAG18 expressed both [FeFe] and [NiFe] hydrogenases) and similarities (both expressed bifurcating type A3 [FeFe] hydrogenases) within the syntrophic bacteria. [FeFe] hydrogenases are typically involved in H_2_ production, whereas [NiFe] hydrogenases tend to be H_2_-consuming, although with many exceptions, and several known hydrogenases also operate in a reversible manner (Lubitz et al., 2014). Furthermore, biochemical characterization and detailed insights into structure and reaction mechanisms of hydrogenases from most syntrophic bacteria are lacking. The functional impact in syntrophic culture of the multiple bifurcating hydrogenases produced by the SPOB candidate MAG15, compared with use of only one group of [FeFe] and higher dependence on [NiFe] hydrogenases by the SAOB *S. schinkii* MAG18, is a possible topic for future research.

For formate formation, the SPOB candidate MAG15 expressed a periplasmic NAD^+^/NADP^+^ utilizing formate dehydrogenase, a NADP^+^-dependent formate dehydrogenase in an operon flanked by genes encoding NADH:ubiquinone oxidoreductase (nuoEF) and formate transporter (fdhC). Nuo is an enzyme complex that transfers electrons from NADH to a quinone pool coupled with proton translocation, where nuoEF forms the NADH binding site (Moparthi and Hägerhäll, 2011). Additional subunits of the Nuo complex (nuoE, F, G, M) were located at other genomic regions in MAG15. A coenzyme F_420_-dependent formate dehydrogenase was also expressed, together with heterodisulphide reductase (hdrA) and F_420_-non-reducing hydrogenase (mvhD) in MAG15. For production of formate during acetate oxidation, *S. schinkii* MAG18 expressed a similar genomic region to that found in MAG15, containing a coenzyme F_420_-dependent formate dehydrogenase-mvhD-hdrABC complex adjacent to a periplasmic formate dehydrogenase (NAD^+^/NADP^+^, Supplementary Figure S8). In addition, the SAOB MAG18 expressed a NADP^+^-reducing formate dehydrogenase next to genes encoding a molybdate uptake system (mobABC) and a F-type ATPase. Formate cannot passively move over the membrane and several copies of formate transporters (fdhC) were expressed in both the SAOB MAG18 and the SPOB candidate MAG15 (Supplementary Note 2). MAG15 also expressed an oxalate/formate antiporter in close genomic proximity to a formate dehydrogenase (fdh). Of particular interest was a non-annotated gene (Supplementary Data SE6, Locus: BGEMGI_15380) found in the nuoEF-fdhC-fdh containing operon of the SPOB MAG15. This gene was among the most highly expressed genes at both extraction time points in the propionate culture and was predicted to be transmembranal (two passes), containing a bacterial OB-fold domain. Bacterial OB-fold domains locate periplasmically and can function as protein membrane anchors (Ginalski et al., 2004).

### The methanogen “*Candidatus* Methanoculleus ammoniitolerans” (MAG17)

MAG17 (90.7% completeness) was taxonomically assigned to the genus *Methanoculleus* (Supplementary Tables S1, S2). The phylogenetic analysis using *Methanoculleus* genomes available at NCBI demonstrated greatest similarity with an uncharacterized *Methanoculleus* species (GCA_013201385.1) (ANI 98%, dDDH 85%; Supplementary Figure S10, Supplementary Table S1) previously identified in the high-ammonia CSTR from which the inoculum used in this study originated (Singh et al., 2021). Based on the genome similarity measurements and the fact that both MAGs were sampled from the same biological source, it is highly likely that the two MAGs represent the same species. However, for the MAG obtained in the previous study, the MAG quality was not sufficiently high for the proposal of a provisional name for this species (Singh et al., 2021).

The second closest species was “*Candidatus* Methanoculleus thermohydrogenotrophicum” (GCA_001512375.1), where the genome comparison revealed values (ANI 83%, DDH of 28%) well below the suggested threshold for species delineation (Goris et al., 2007; Jain et al., 2018). MAG17 had genome size 2.68 Mbp, 58.4% GC content and 3,344 protein coding sequences, which is comparable to the type strain *M. bourgensis* MS_2_T (2.79 Mbp, 60.6% GC content, 2,586 protein coding sequences) (Maus et al., 2015). However, the 25% smaller genome size of the closest neighbor “*Ca.* M. thermohydrogenotrophicum” (2.15 Mbp, 59.5% GC content, 2,415 protein coding sequences) indicates that part of that genome is missing, bringing some uncertainties to the comparison of values against that metagenome. Based on the high quality of MAG17 and the genome comparison suggesting that MAG17 [and GCA_013201385.1 (Singh et al., 2021)] will form a novel species when isolated, we propose the provisional name “*Candidatus* Methanoculleus ammoniitolerans”.

**Figure 7:**
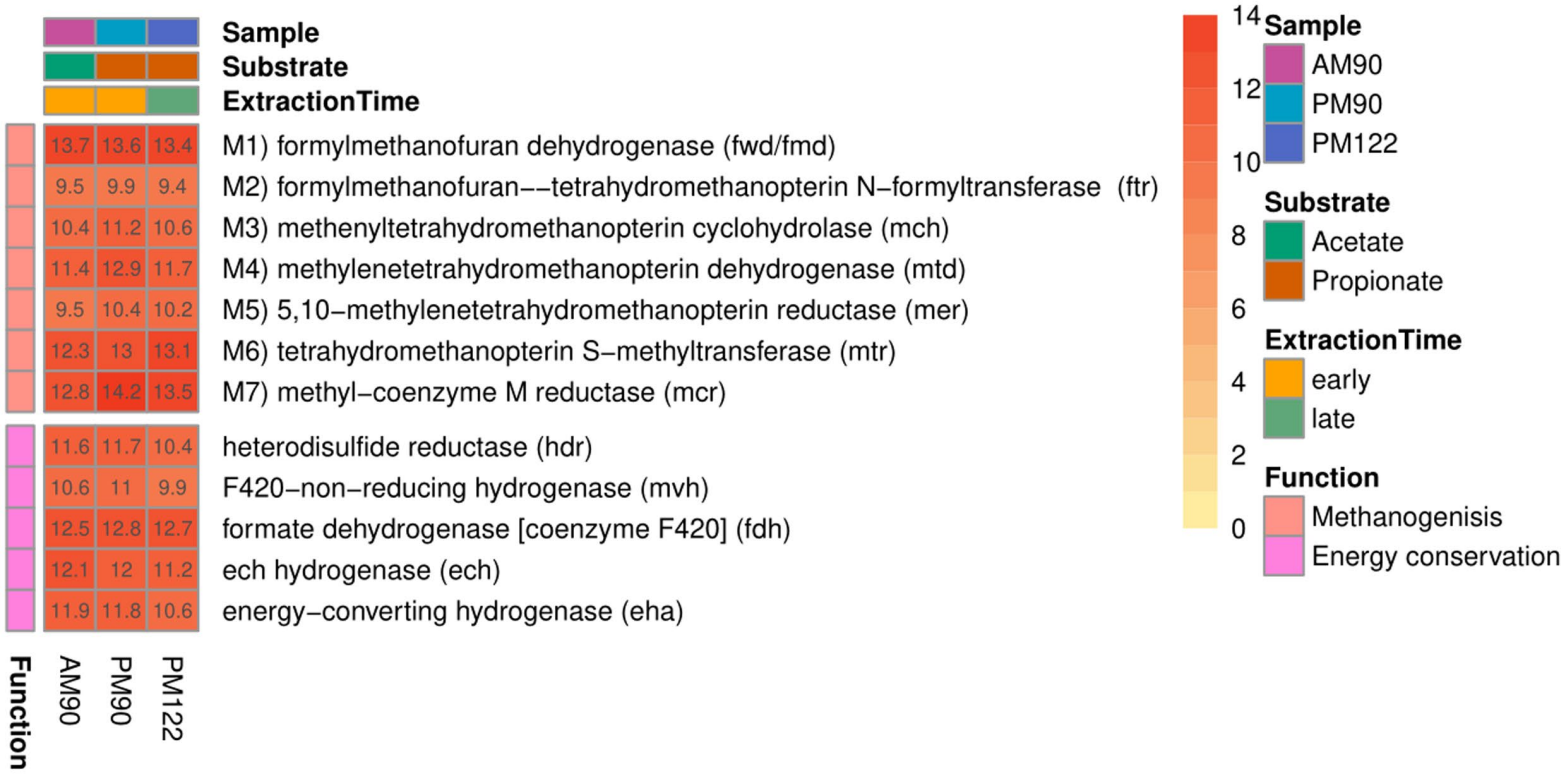
Expression of genes in the hydrogenotrophic pathway by the methanogen “*Candidatus* Methanoculleus ammoniitolerans” (MAG17) in propionate (PM90, PM122) and acetate batch assays (AM90). Heatmap values are aggregated log2-transformed deseq2 normalized count, of all copies and subunits for each respective gene.

**Figure 8:**
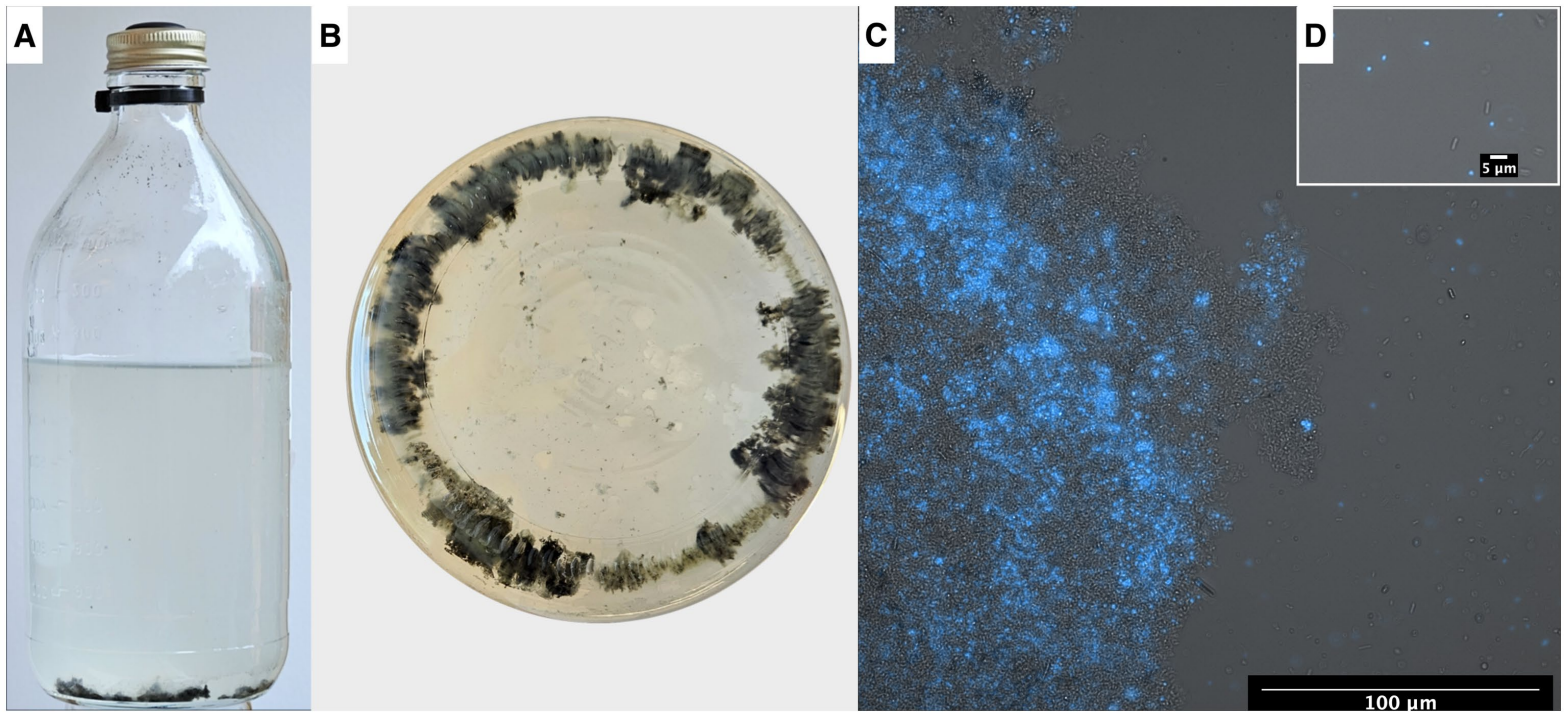
**(A)** Serum bottle containing syntrophic propionate-oxidizing community, **(B)** bottle visualized from below showing the flocs within the microbial community. Fluorescence-brightfield composite micrograph showing autofluorescence of “*Ca.* Methanoculleus ammoniitolerans” **(C)** in floc community and **(D)** as planktonic cells.

All genes needed for hydrogenotrophic methanogenesis were expressed by MAG17 (Figure 7). As reported for *M. bourgensis* MS2^T^ (Maus et al., 2015; Kougias et al., 2017), genes encoding enzymes for steps M1, M2, M6 and M7 (Figures 4, 8) were located in a core-pathway operon in MAG17, whereas the hydrogenotrophic genes for the other steps were scattered throughout the genome. For the first step (M1), involving formylmethanofuran dehydrogenase (Fwd), the reduction of CO2 to formylmethanofuran necessitates reduced ferredoxin. The reduction of ferredoxin is an endergonic reaction and in MAG17 genes encoding Fwd were located at four different loci throughout the genome next to distinct enzymatic systems for reduction of ferredoxin: (i, ii) a membrane-bound [NiFe]-hydrogenase of either type 4e (Ech) or 4 h (Eha) using hydrogen as electron donor and proton or sodium motive force to drive the reaction, respectively; (ii) bifurcative coupling with exergonic reduction of CoM-S-S-CoB using hydrogen as electron donor (Figure 4, Supplementary Data SE6); and (iii) using formate via a membrane-bound formate dehydrogenase (fdhAB). This indicates that MAG17 has flexibility when it comes to the source of reductive power needed to drive the endergonic ferredoxin reduction. This can be advantageous during anabolic growth, since reduced ferredoxin also can be created without coupling to reduction of the heterodisulphide (CoM-S-S-CoB) as part of methanogenesis.

For reduction of coenzyme F_420_ (oxidized in steps M4 and M5), either a cytoplasmic [NiFe] type 3a hydrogenase (Frh) or a membrane-attached formate dehydrogenase was expressed. Comparing the hydrogenase repertoire of MAG17 with that of closely related species (Supplementary Figures S10, S11) revealed that, in contrast to the thermophilic “*Ca.* M. thermohydrogenotrophicum” but similarly to the mesophilic ammonia-tolerant *M. bourgensis*, MAG17 expressed a bifurcating 3c [NiFe] hydrogenase (Supplementary Figure 10). This enzyme is crucial for operating hydrogenotrophic methanogenesis in a circular fashion (i.e., the Wolfe cycle), coupling the reduction of ferredoxin to the reduction of heterodisulphide (CoM-S-S-CoB) (Thauer, 2012). The conjoined expression of Ech and Eha observed for MAG17 in the present study has previously been suggested as a possible explanation for ammonia tolerance in *Methanothermobacter* sp. studied in thermophilic batch reactors (Yan et al., 2020).

In summary, these findings highlight metabolic flexibility in reduction of oxidized electron carriers by “*Ca.* M. ammoniitolerans” MAG17, using both molecular hydrogen and formate as sources of reductive power for the reduction of electron carriers needed for hydrogenotrophic methanogenesis.

Despite absence of alcohol in the cultures studied, alcohol dehydrogenase was expressed by the methanogen, in particular in acetate and early propionate batches. Expression of secondary alcohol dehydrogenases in cultures not supplied with alcohol has been reported previously in a hydrogenotrophic methanogen under H_2_-limited conditions (Widdel and Wolfe, 1989), indicating that deeper insights could be gained by further study of the role of this protein in hydrogenotrophic methanogens. *Methanoculleus* sp. use minor amounts of acetate for biosynthesis (Schnürer et al., 1999; Maus et al., 2015), and MAG17 expressed Acetyl-CoA synthetase/acetate-CoA ligase (Supplementary Figure 12), which uses ATP for activation of acetate to acetyl-CoA.

Microscopic analyses of the enrichment culture demonstrated presence of cells with autofluorescence in the 420 nm region. The “*Ca.* Methanoculleus ammoniitolerans” cells were identified as irregular cocci with diameter 1–2 μm, with limited motility, and often residing as part of large cellular aggregates (Figure 8C).

### Potential function of Acetomicrobium sp. (MAG16) in the syntrophic communities

Since 4.7% of the transcriptomic reads mapped against MAG16 in this study (Supplementary Figure S6), detailed analysis of this MAG was conducted. The results showed that MAG16 belonged to the genus *Acetomicrobium* (class Synergistia, order Synergistales, family Acetomicrobiaceae) (Supplementary Table S1). The closest characterized species to MAG16 was found to be *Acetomicrobium mobile* (ANI 98%) (Supplementary Figure S13) isolated from an anaerobic lagoon treating wool-scouring wastewater (Menes and Muxí, 2002). The *Acetomicrobium* sp. MAG16 showed similar gene expression level in both the acetate-fed and propionate-fed batches (Supplementary Figure S12). The bacterium lacked key genes needed for the WL pathway, such as acetyl-CoA synthetase and carbon monoxide dehydrogenase, and the complete gene set needed for initial activation of acetate (expression of acetate kinase, but no phosphotransacetylase or an acetyl-CoA synthetase) (Supplementary Figure S12). However, MAG16 expressed genes involved in the reductive glycine pathway. In this CO_2_ fixation pathway, CO_2_ is reduced to formate and through additional condensation of CO_2_ forming intermediates such as glycine, acetyl-P and acetyl-CoA (Sánchez-Andrea et al., 2020). The species expressed all genes needed for the glycine cleavage system except for serine dehydratase, although a threonine dehydratase with similar catalytic function was expressed. The reductive glycine pathway has been proposed to be operated in the oxidative direction (Sánchez-Andrea et al., 2020), in conjunction with the methyl branch of WLP, acting as a hypothesized acetate oxidation pathway, thus competing with the SAOB for available acetate (Zhu et al., 2020; Li et al., 2022; Kieft et al., 2023). However, considering the lack of experimental evidence, this should be viewed as speculative. Since a species belonging to the genus *Acetomicrobium* with a similar activity profile has been observed in a thermophilic syntrophic propionate and acetate-oxidizing culture enriched under high-ammonia conditions in our laboratory (Singh et al., 2023), its activity naturally piqued our interest. Given the sustained activity observed in both acetate and propionate-fed cultures over time, it is conceivable that this species participates in the degradation of either acetate or utilizes formate as a growth substrate. However, considering the wide range of substrates used by members of Acetomicrobium, including organic acids, sugars, amino acids including cysteine (by some members), this bacterium might ferment compounds included in the yeast extract, as well as compounds released from dead cells or possibly grew oxidatively using cysteine as electron acceptor.

### Expression of genes potentially related to ammonia tolerance differed between the syntrophic bacteria and the methanogen

**Figure 9:**
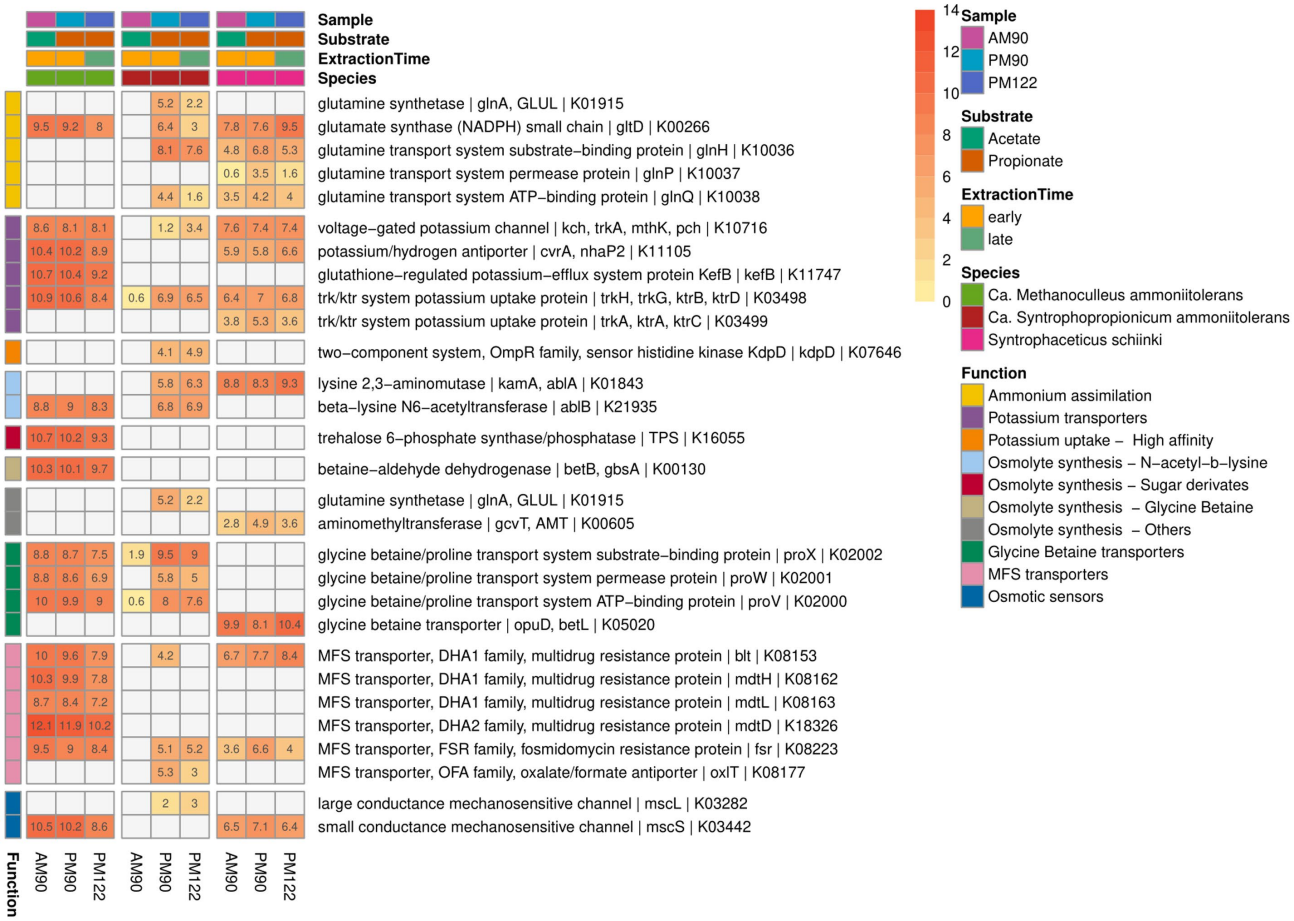
Gene expression potentially related to ammonia tolerance activity by the syntrophic propionate-oxidizing bacteria (SPOB) “*Ca.* Syntrophopropionicum ammoniitolerans” (MAG15), the syntrophic acetate-oxidizing bacteria (SAOB) *S. schinkii* (MAG18) and the methanogen “*Ca.* Methanoculleus ammoniitolerans” (MAG17) sampled from propionate and acetate batch assays (AM90, PM90, PM122). Heatmap values are the aggregated log2-transformed deseq2 normalized count, of all copies of each respective gene. Genes not expressed are shown in gray. Annotation of rows is based on KEGG annotations and follows the order: Gene name | Protein Symbol | KO identifier.

As the enrichment cultures were cultivated under high-ammonia (Supplementary Figure S14) conditions and originated from CSTRs operated in high-ammonia conditions, the metatranscriptomic data were used to identify expression of genes known to be involved in microbial strategies to cope with elevated ammonia and the associated osmotic stress (Figure 9). Common strategies to counteract osmotic stress include uptake of cations and synthesis or uptake of compatible solutes, where the latter are low molecular weight organic compounds that accumulate in the cytoplasm to balance the external osmotic stress (Martin et al., 1999; Sleator and Hill, 2002). Expression by the SPOB (MAG15) indicating synthesis of compatible solutes included the genes for lysine 2,3-aminomutase (ablA) and beta-lysine N6-acetyltransferase (ablB) for conversion of lysine to the compatible solute N^ε^-acetyl-β-lysine first characterized in methanogenic archaea (Sowers et al., 1990). Interestingly, these genes were located adjacent to genes encoding a mechanosensitive channel (ybioO) previously shown to protect *E. coli* cells when exposed to hyperosmotic stress (Edwards et al., 2012). In contrast, the methanogen (MAG17) only expressed the ablB gene and the SAOB (MAG18) only expressed the ablA gene. Furthermore, MAG17 expressed the gene trehalose 6-phosphate synthase for synthesis of the glucose-derived compatible solute trehalose. This compound has been shown to accumulate in members of the domain *Archaea* in response to osmotic stress and genes for trehalose synthase have also been identified in several *Methanoculleus* genomes (Empadinhas and da Costa, 2006; Maus et al., 2015; Chen et al., 2019).

Compatible solutes can also be taken up from the extracellular environment. Both the syntrophic bacteria and the methanogen expressed glycine betaine/proline ABC transporters (Figure 9). Intriguingly, neither of the syntrophic partners nor *Acetomicrobium* sp. (MAG16) had the complete set of genes needed for synthesis of glycine betaine, but all expressed genes needed for the uptake systems (Figure 9, Supplementary Data SE6). It is possible that the compatible solute was synthesized by one of the other species in the culture, despite overall low relative expression level (Yan et al., 2020, 2022).

A common initial response to osmotic stress involves uptake of potassium ions. All three species (MAG15, MAG17, MAG18) expressed the gene for low-affinity potassium uptake protein (trk), but lacked ATP-consuming, high-affinity uptake variants (Sleator and Hill, 2002). None of the organisms expressed ammonia transporters, a trait that can be beneficial to withstand the stress in ammonia-rich environments (Maus et al., 2015; Manzoor et al., 2016b; Singh et al., 2021). Numerous different chaperones (DnaK, DnaJ, archaeal chaperonin, ClpB, ClpC, ClpX) and heat shock proteins (GroEL, HSP20) were expressed by the syntrophic community (Supplementary Figure S15), many of which were coded for by the most highly expressed genes. Chaperones are a common microbial stress response (Hill et al., 2002; Papadimitriou et al., 2016) and high expression of chaperones by the syntrophs could provide protection against protein denaturation and potentially be fundamental to the ability of syntrophic communities to deal with ammonia stress. However, it could also be an artifact of the stress induced during RNA extraction. To gain a deeper understanding of how these syntrophic communities manage ammonia stress, it is imperative to conduct future studies specifically focused on investigating the microbial response to such stressors.

### Gene expression of potential importance for mobility, flocculation or interspecies cooperation

**Figure 10:**
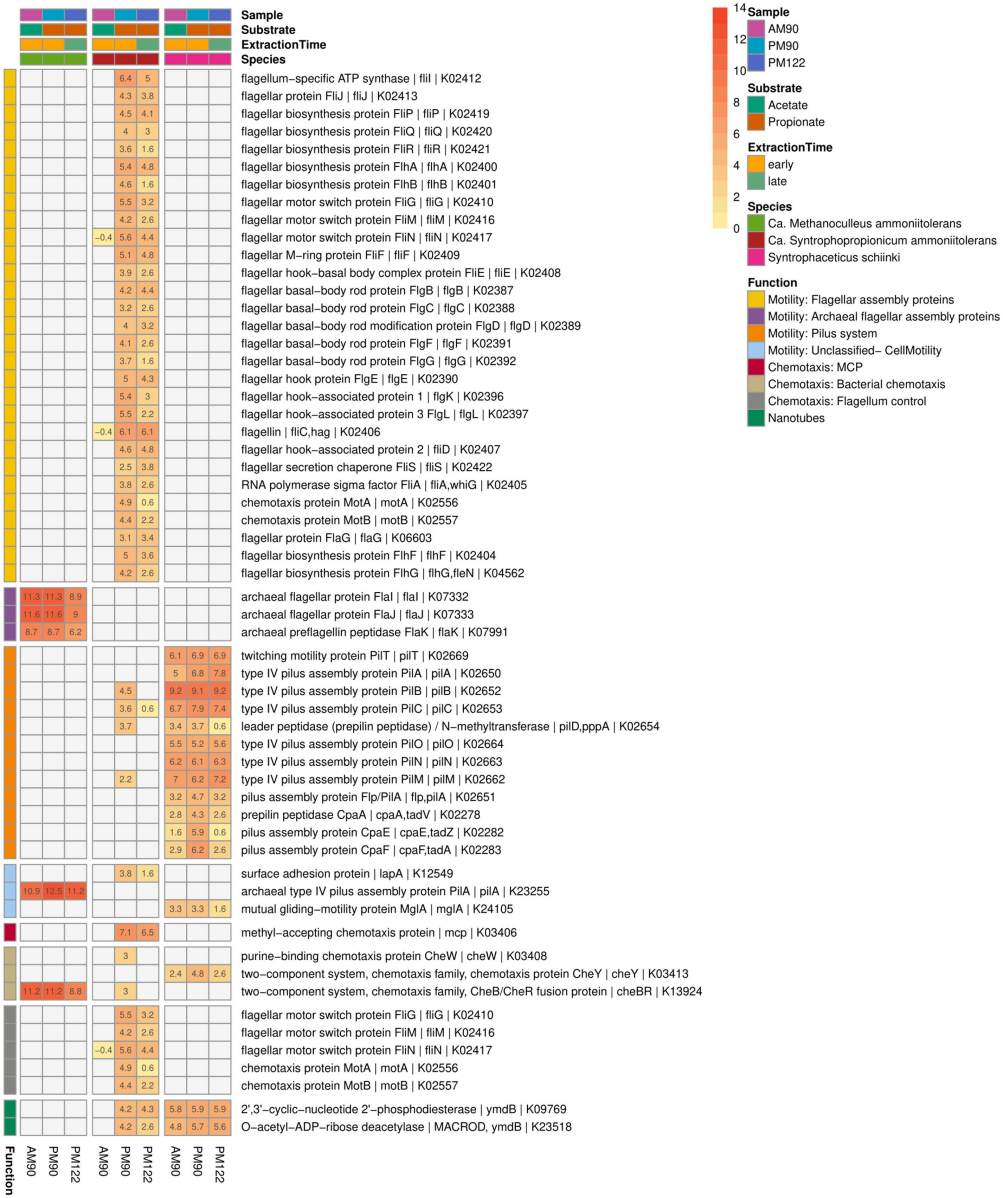
Expression of genes potentially related to mobility, biofilm formation, cell contact and interspecies cooperation activity by the syntrophic propionate-oxidizing bacteria (SPOB) “*Ca.* Syntrophopropionicum ammoniitolerans” (MAG15), the syntrophic acetate-oxidizing bacteria (SAOB) *S. schinkii* (MAG18) and the methanogen “*Ca.* Methanoculleus ammoniitolerans” (MAG17) in propionate and acetate batch assays (AM90, PM90, PM122). Heatmap values are shown as log2 of the deseq2 normalized raw counts, where genes with identical KO identifiers are merged. Genes not expressed are shown in gray. Annotation of rows is based on KEGG annotations and follows the order: Gene name | Protein Symbol | KO identifier.

Visual inspection of the syntrophic cultures revealed formation of large flocs during the exponential phase of VFA degradation in both the acetate- and propionate-oxidizing cultures (Figure 10). Hence, a specific search was made for gene expression related to microbial aggregation and interaction. Flagella and pili are known to be used for mobility, but have also been shown to facilitate biofilm formation, cell contact and interspecies cooperation. For instance, the propionate oxidizer *P. thermopropionicum* has been demonstrated to initiate contact with its methanogenic partner through the flagellar cap protein FliD (Shimoyama et al., 2009). The SPOB candidate MAG15 expressed genes encoding a complete flagellum complex, but also expressed pilus-associated genes (pilB, pilC, pilD, pilM) during early propionate degradation, albeit at low levels.

In contrast to the SPOB (MAG15), the SAOB (MAG18) completely lacked flagella-associated genes (Figure 10) but instead expressed pilus genes, especially subunits encoding the motor complex (pilB, pilC, pilT) (Hospenthal et al., 2017), indicating its involvement in motility. Notably, the subunits involved in formation of the secretin complex (pilQ) and alignment complex (pilP) were not present in the genome. The SAOB MAG18 also expressed Flp-type prepilin, which was organized in a characteristic operon containing tight adherence (tad) genes in close proximity. Flp-type pili have been shown to be involved in adherence, twitching motility, DNA uptake and biofilm formation (Cai et al., 2021). The methanogen MAG17 had a limited set of archaeal flagellar genes (flaI, flaJ, flaK), but lacked other critical archaeal flagellar genes needed for motility. The methanogen also expressed genes for archaeal type IV pilus assembly proteins (pilA), some of which resided on an operon together with the aforementioned archaeal flagellar genes found in the genome.

As regards chemotaxis and orchestration of the motility machinery, the SPOB MAG15 expressed genes for methyl-accepting chemotaxis proteins used for recognition of extracellular stimuli, but lacked further signal-transducing genes needed for regulation of flagellar activity (Wadhams and Armitage, 2004) (Figure 10). Although quorum sensing systems have been shown to play an important role in some syntrophic systems (Yin et al., 2020; Doloman et al., 2024) no clear indication of complete quorum sensing machinery was observed based on the KEGG reference pathway analysis for any of the three species.

Both the SAOB and the SPOB expressed genes for phosphodiesterase (ymdB), previously shown to be essential for the formation of nanotubes (small membranous structures that allow for exchange of nutrients and genetic material between cells), which are important for the switch from motile to attached sessile lifestyle in *Bacillus subtilis* (Diethmaier et al., 2011; Dubey et al., 2016). There was a striking similarity between the operon harboring ymdB in the SAOB and the SPOB. The gene was next to that for a 167 aa long hypothetical protein, a stage V sporulation protein S (spoVS) and numerous other shared proteins, including a vesicle-fusing ATPase. This is consistent with findings in a previous study of a thermophilic and anaerobic digester-derived enrichment culture of expression of ymdB by the ammonia-tolerant SPOB and SAOB candidates, indicating that this activity plays an important role in syntrophic cultures at high ammonia levels (Singh et al., 2023). Furthermore, the SPOB MAG15 expressed genes for a surface adhesion protein (lapA), which has been shown to be a key factor in biofilm formation (Ainelo et al., 2017).

## Conclusion

This study elucidates the energetic pathways utilized by the mesophilic SPOB candidate “*Ca. S. ammoniitolerans*”, the SAOB *S. schinkii* and a novel hydrogenotrophic methanogenic candidate “*Ca.* M. ammoniitolerans” during syntrophic propionate oxidation at high ammonia conditions. Specifically, the study reveals that the both acid oxidizers share the same methanogenic partner, and that the gene expression by the SAOB and the hydrogenotrophic methanogen was not affected by presence of the SPOB. The SPOB exclusively expressed [FeFe]-type hydrogenases, while the SAOB expressed both [FeFe] and [NiFe] hydrogenases. Both species also expressed bifurcating type A3 [FeFe] hydrogenases, and similar regions encoding formate dehydrogenases. The methanogen expressed a bifurcating 3c [NiFe] hydrogenase, suggesting the operation of hydrogenotrophic methanogenesis in a circular manner (i.e., the Wolfe cycle). Additionally, the concurrent expression of membrane-bound [NiFe]-hydrogenase Ech and Eha by the methanogen reinforces previous association with ammonia tolerance. Furthermore, gene expression profiles by the three syntrophic microorganisms indicated various mechanisms proposed to mitigate ammonia inhibition, including synthesis of compatible solutes or their uptake from the surrounding environment. Further research should be conducted to validate these findings. Floc formation was observed during growth and was postulated to occur as a stress response to high ammonia level or as an act by the cells to foster proximity to the cooperating partner. Gene expression potentially related to flocculating activity included flagella- (“*Ca.* S. ammoniitolerans”) and pili-associated (*S. schinkii*, “*Ca.* M. ammoniitolerans”) genes and genes encoding proteins previously shown to be essential for formation of nanotubes (“*Ca.* S. ammoniitolerans”, *S. schinkii*), a trait shared with thermophilic ammonia-tolerant syntrophic propionate- and acetate-oxidizing bacteria.

## Data availability statement

The datasets presented in this study can be found in online repositories. The names of the repository and accession number(s) can be found at: NCBI repository (https://www.ncbi.nlm.nih.gov/), accession numbers: PRJNA1016136, PRJNA1016271, PRJNA1016301, JAVTVG000000000, JAVTVF010000000, JAVTVH010000000, JAVTVI010000000.

## Author contributions

NW: Writing – original draft, Data curation, Investigation, Methodology, Software, Visualization. AS: Writing – review & editing, Data curation, Software. JO: Writing – review & editing, Visualization. JD: Writing – review & editing, Formal analysis, Data curation. MW: Writing – review & editing, Conceptualization, Formal analysis, Funding acquisition, Investigation, Methodology, Project administration, Resources, Supervision, Validation.
