# Supplementary material for: Catabolism and interactions of syntrophic propionate- and acetate oxidizing microorganisms under mesophilic, high-ammonia conditions
Source: Front Microbiol. 2024 Jun 5;15:1389257. doi: 10.3389/fmicb.2024.1389257 (PMC11201294; doi:10.3389/fmicb.2024.1389257)
Supplement: Supplementary file 1 [file Data_Sheet_1.ZIP › SUP/Supplementary Material.docx]

***Supplementary Material***

**1 Supplementary Figures**


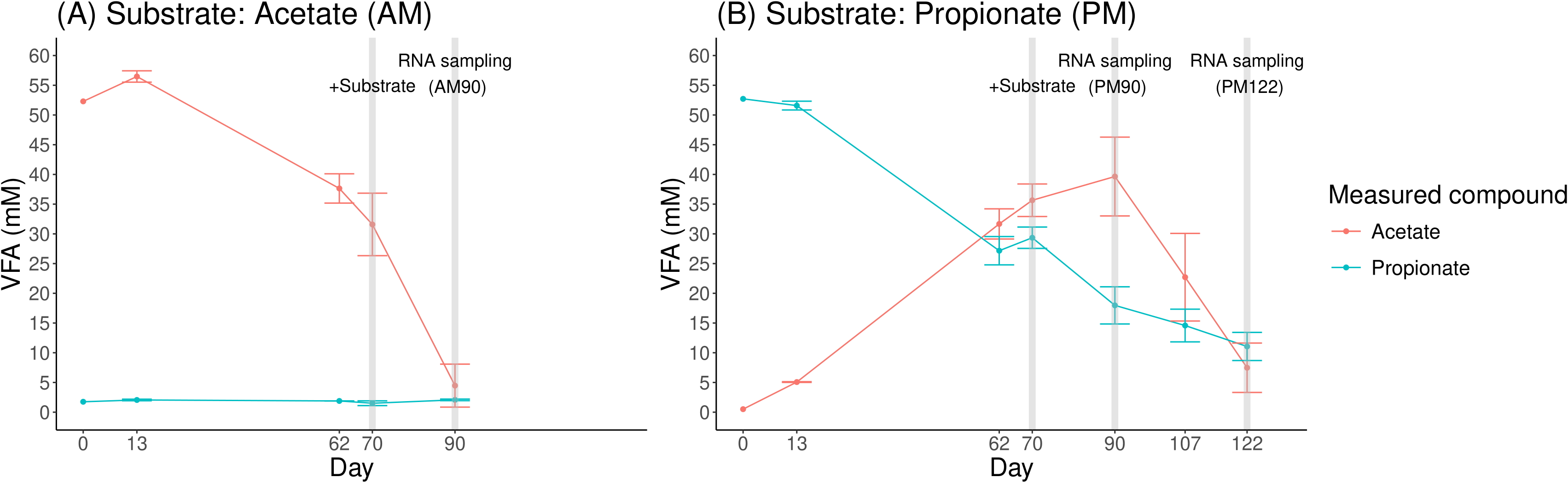


**Fig. S1** Volatile fatty acid (VFA) concentration (acetate/propionate) and RNA extraction time points for meta-omics cultures (AM/PM) supplied with either A) acetate (AM) or B) propionate (PM). Both cultures were spiked with substrate at day 70 to reach the level of cell biomass required for RNA extraction. All cultures were sampled for RNA extraction at day 90 (AM90, PM90) and the PM cultures were also sampled on day 122 (PM122) to ensure simultaneous acid conversion of both acid oxidizers. AM: Propionate enrichment culture, conducted in quadruplicates, fed 50 mM acetate used for metagenomic and metatranscriptomic analysis. PM: Propionate enrichment culture, conducted in quadruplicates, fed 50 mM propionate used for metagenomic and metatranscriptomic analysis.


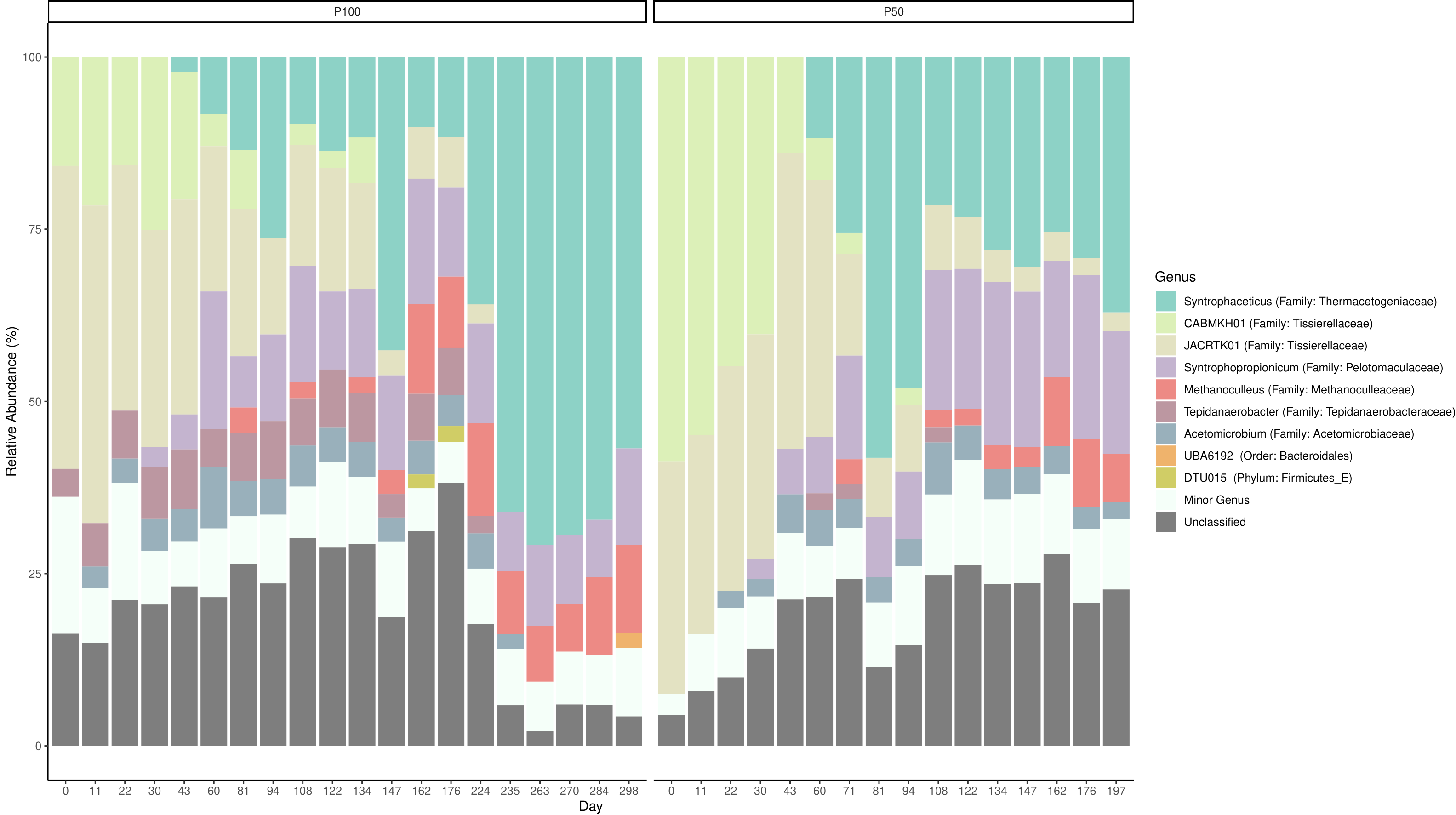


**Fig. S2** Percentage relative abundance at genus level for degradation dynamics and 16S microbial abundance batch assays supplied 50 mM (P50) and 100 mM (P100) propionate. Abundance was determined by 16S rRNA gene sequencing over the course of degradation and is reported as the mean relative abundance of biological replicates. Genera with less than (>2%) relative abundance are shown as “Minor Genus” and amplicon sequencing variants lacking taxonomical assignment at a genus level are shown as “Unclassified”. The taxonomical classification of groups with non-valid nomenclature are shown in brackets. For each setting, triplicate batch assays were conducted.


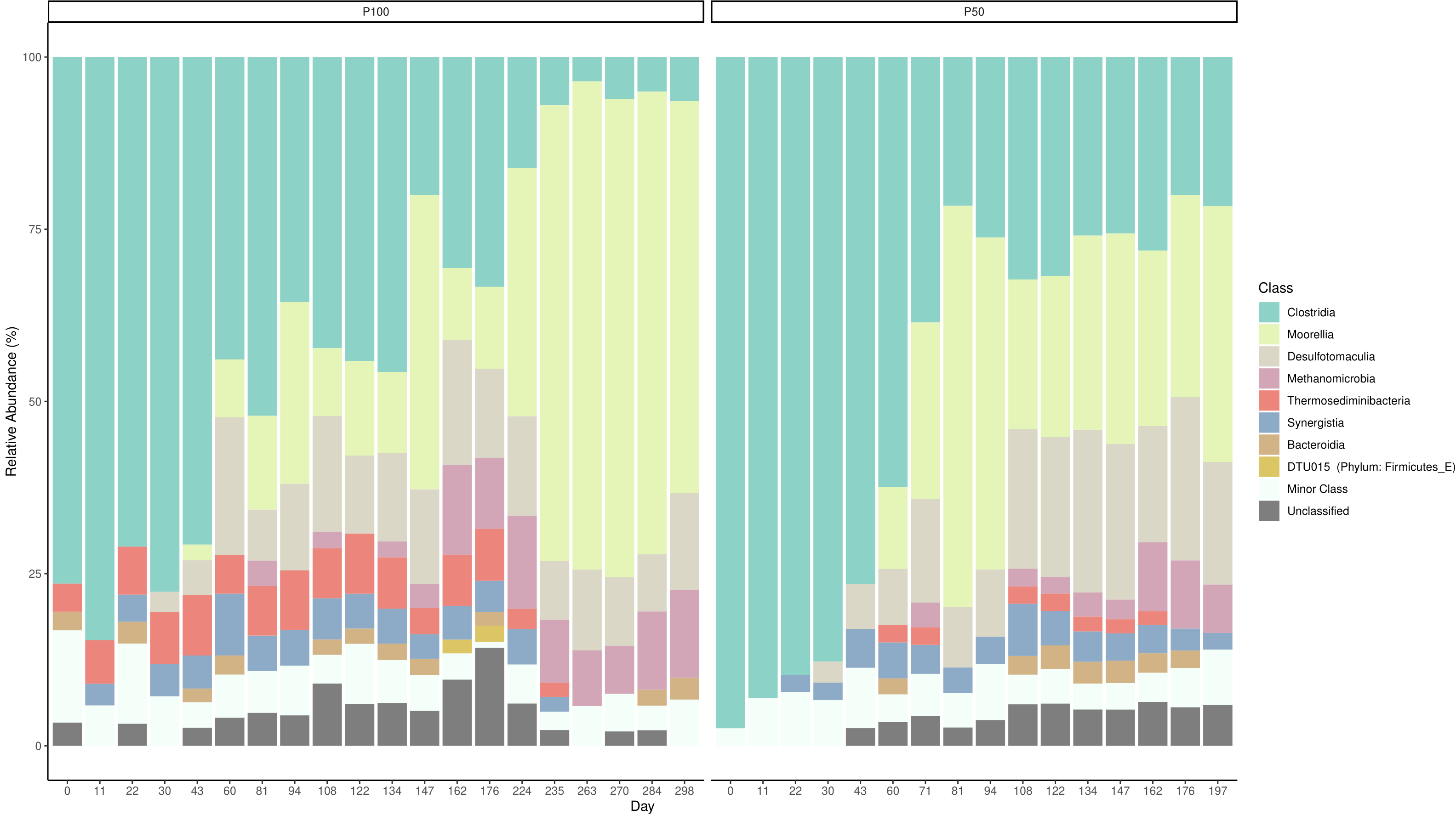


**Fig. S3** Percentage relative abundance on class level (>2%) as determined by 16S rRNA gene sequencing over the course of degradation in batches initiated at 50 mM (P50) and 100 mM propionate (P100). The taxonomical classification of groups with non-valid nomenclature are shown in brackets. For each setting, triplicate batch assays were conducted.


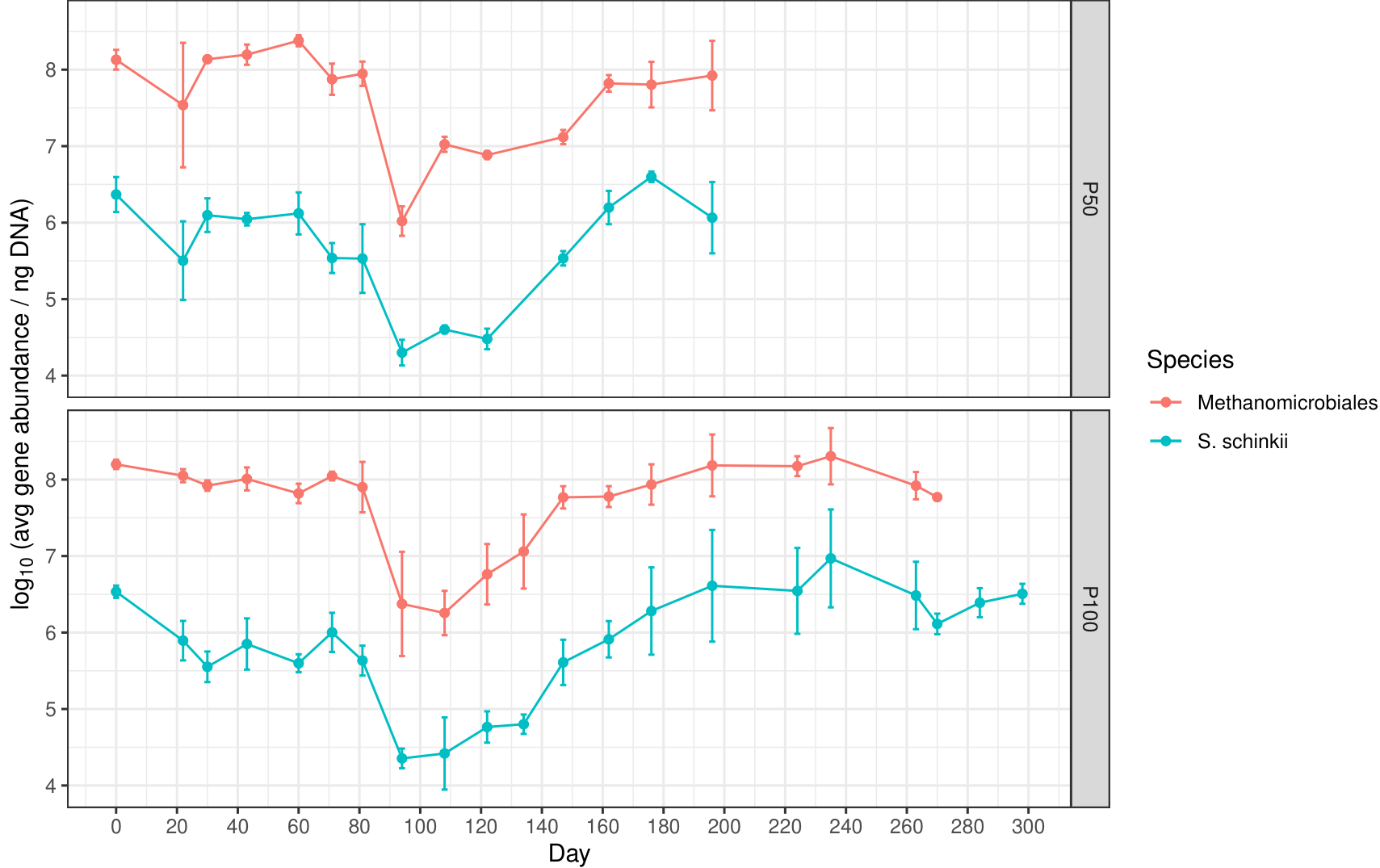


**Fig. S4** Average logarithmic gene abundance of 16S rRNA genes of members of Methanomicrobiales and the syntrophic acetate oxidizing bacteria *Syntrophaceticus schinkii* in batches initiated at 50 mM (P50) and 100 mM propionate (P100). The gene abundance was determined using quantitative PCR (qPCR). For each setting, triplicate batch assays were conducted.


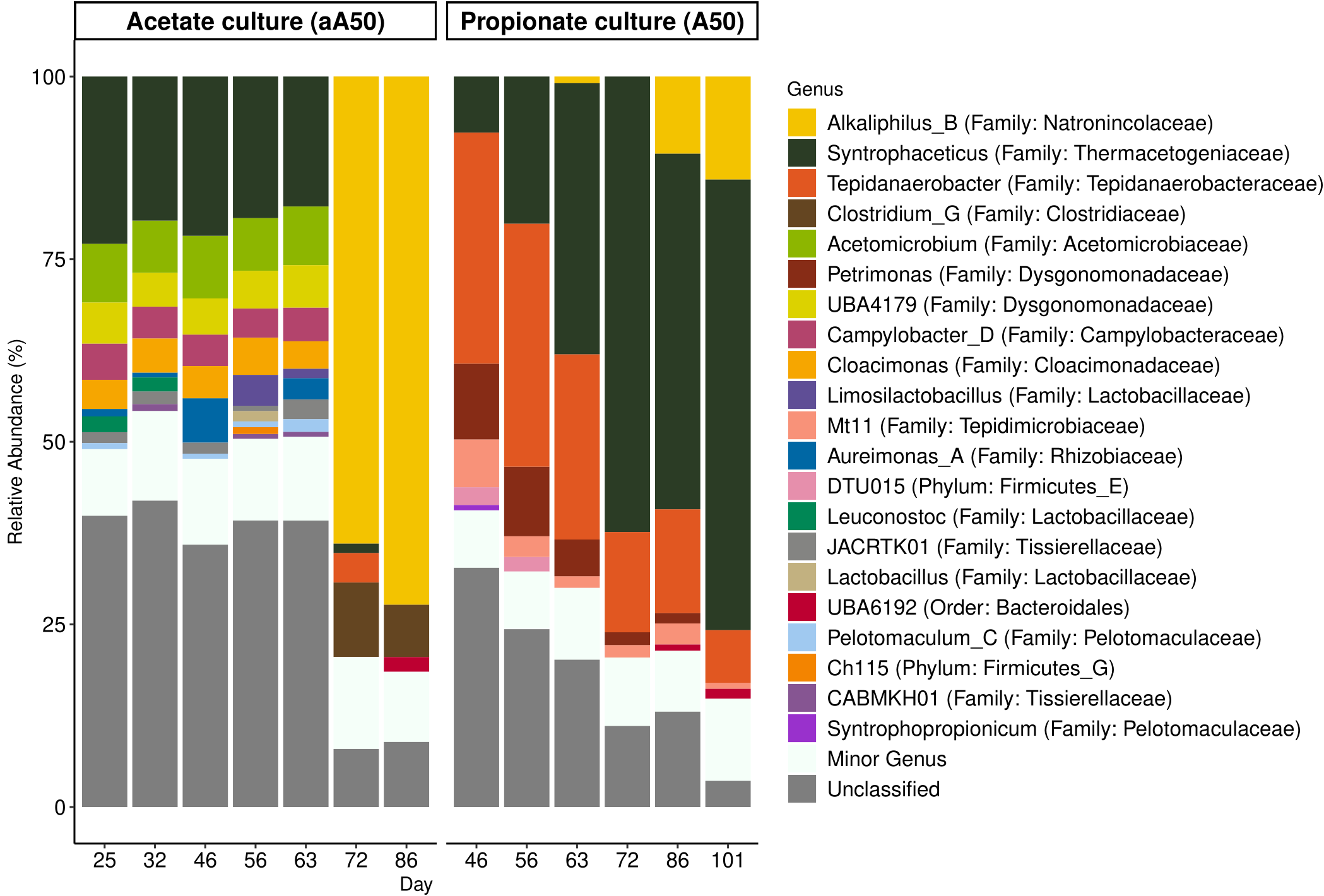


**Fig. S5** Percentage relative abundance on genus level (>2%) as determined by 16S rRNA gene sequencing over the course of degradation in batches initiated at 50 mM acetate and inoculated with either acetate enrichment culture (aA50) or propionate enrichment culture (A50). The taxonomical classification of groups with non-valid nomenclature are shown in brackets For each setting, triplicate batch assays were conducted.


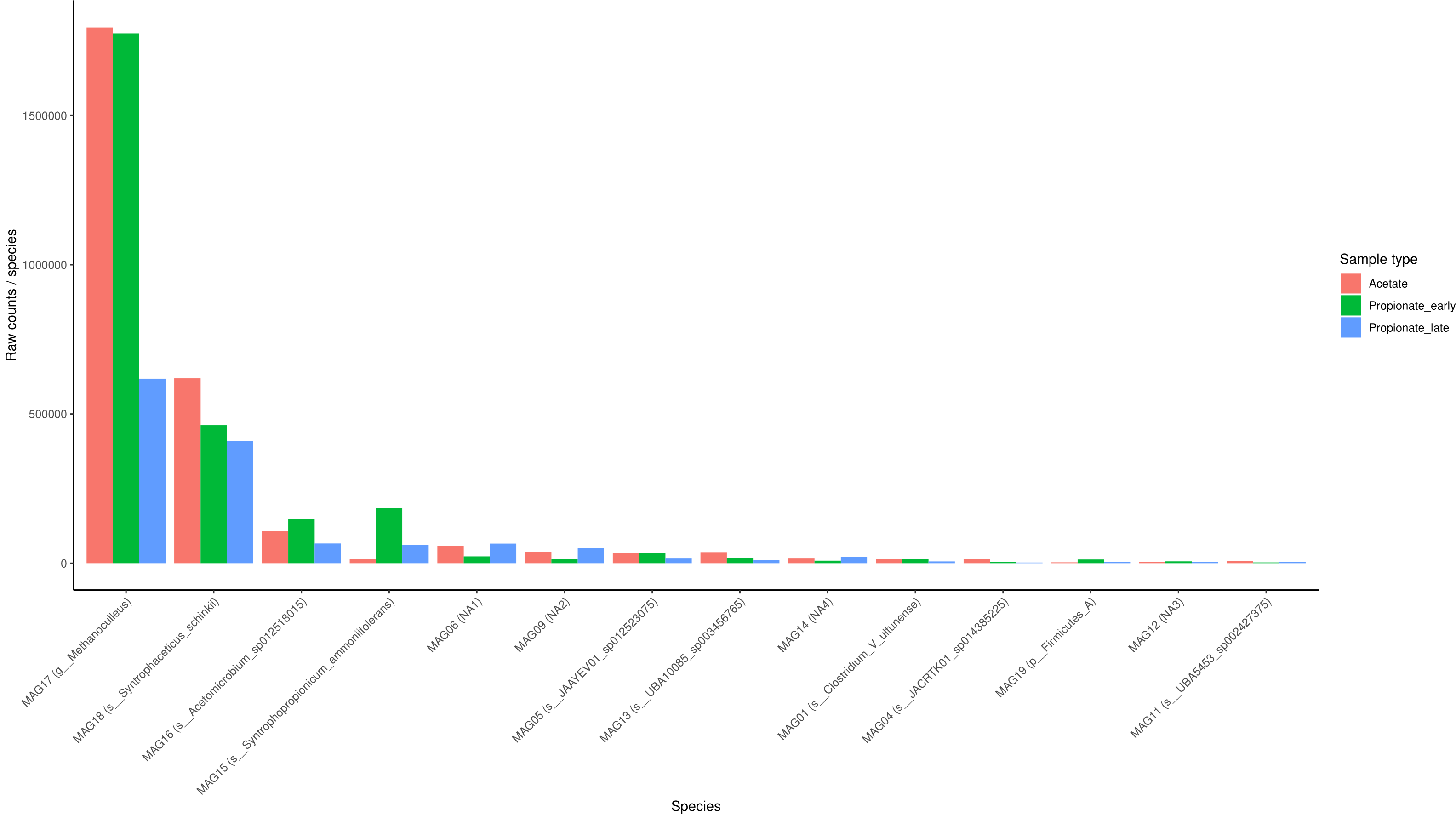


**Fig. S6** Comparison of transcript abundance in metatranscriptomic samples from the acetate-fed (AM90) and two propionate-fed batches at early (PM90) and later stage (PM122) of propionate degradation of all retrieved metagenomic assembled genomes. Results are shown as total raw count per species.





**Fig. S7** Phylogenetic relationships of ‘*Candidatus* Syntrophopropionicum ammoniitolerans’ (MAG15) with known or putative syntrophic propionate-oxidizing bacteria genomes available at NCBI.


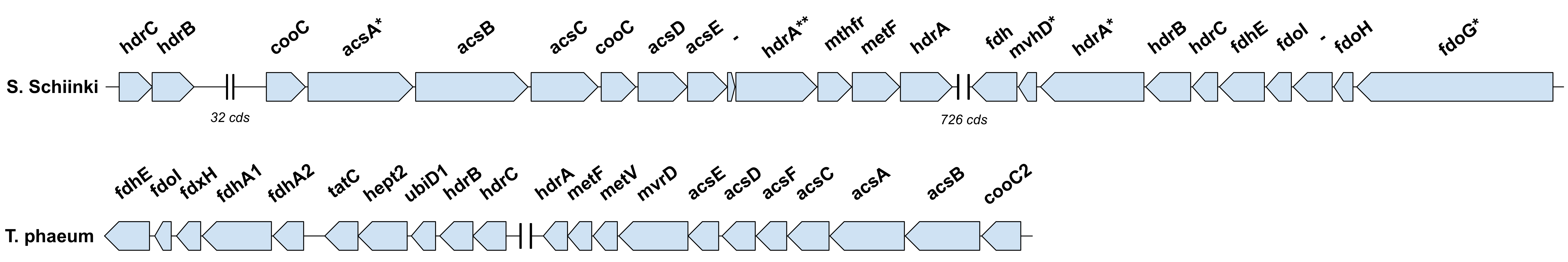


**Fig. S8** Organization of the gene cluster containing methylenetetrahydrofolate reductase (MTFR) and heterodisulphide reductase (*Hdr*) for the SAOB *Syntrophaceticus schinkii* (*S*. *schinkii*, MAG18) and *Thermacetogenium phaeum* (*T. phaeum*). The gene organisation of *T. phaeum* is co-adapted from (Keller et al., 2019). Genes included in the CODH complex are: *cooC*; CO dehydrogenase maturation factor, *cooS*, *acsA*; anaerobic carbon-monoxide dehydrogenase catalytic subunit, *cdhE*, *acsC*; acetyl-CoA decarbonylase/synthase, *CODH*/*ACS* complex subunit gamma, *acsE*; 5-methyltetrahydrofolate corrinoid/iron sulphur protein methyltransferase. *Denotes sequential coding sequences with identical annotation, which have been merged for better visualization, - denotes hypothetical proteins.


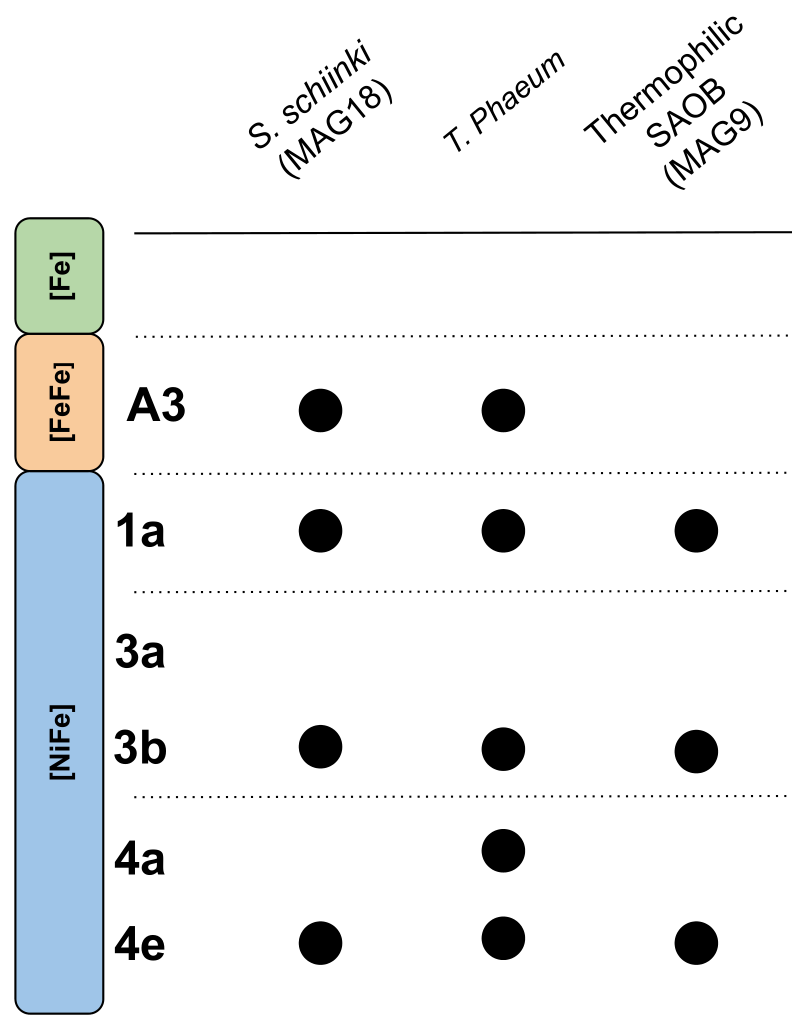


**Fig. S9** Presence of hydrogenases in *Syntrophaceticus schinkii* (MAG18*), Thermacetogenium phaeum* and an ammonia-tolerant thermophilic SAOB (Singh et al., 2023).


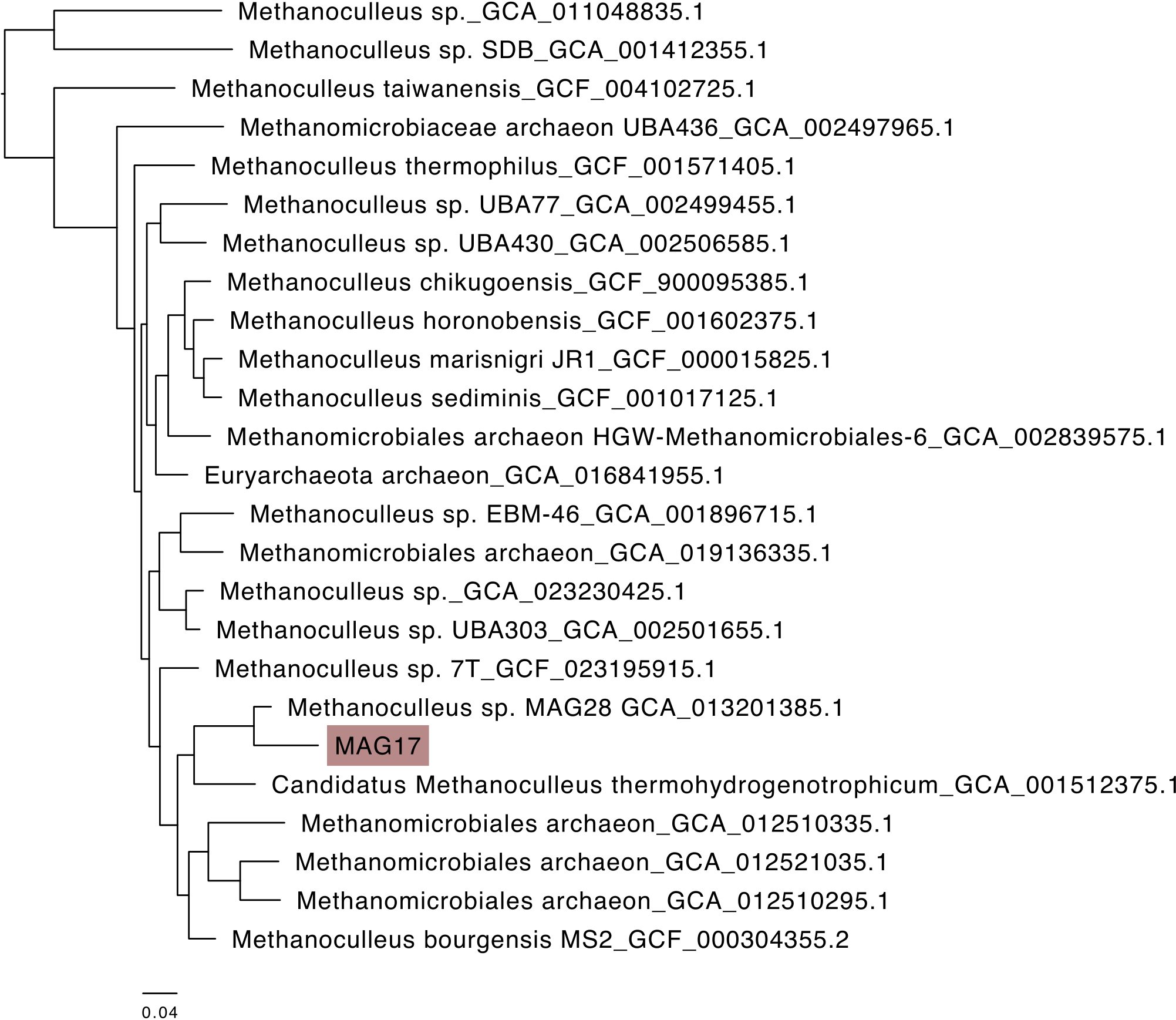


**Fig. S10** Species tree based on orthologous sequences of ‘*Candidatus* Methanoculleus ammoniitolerans’ MAG17 and other species and candidates in the genus *Methanoculleus*.


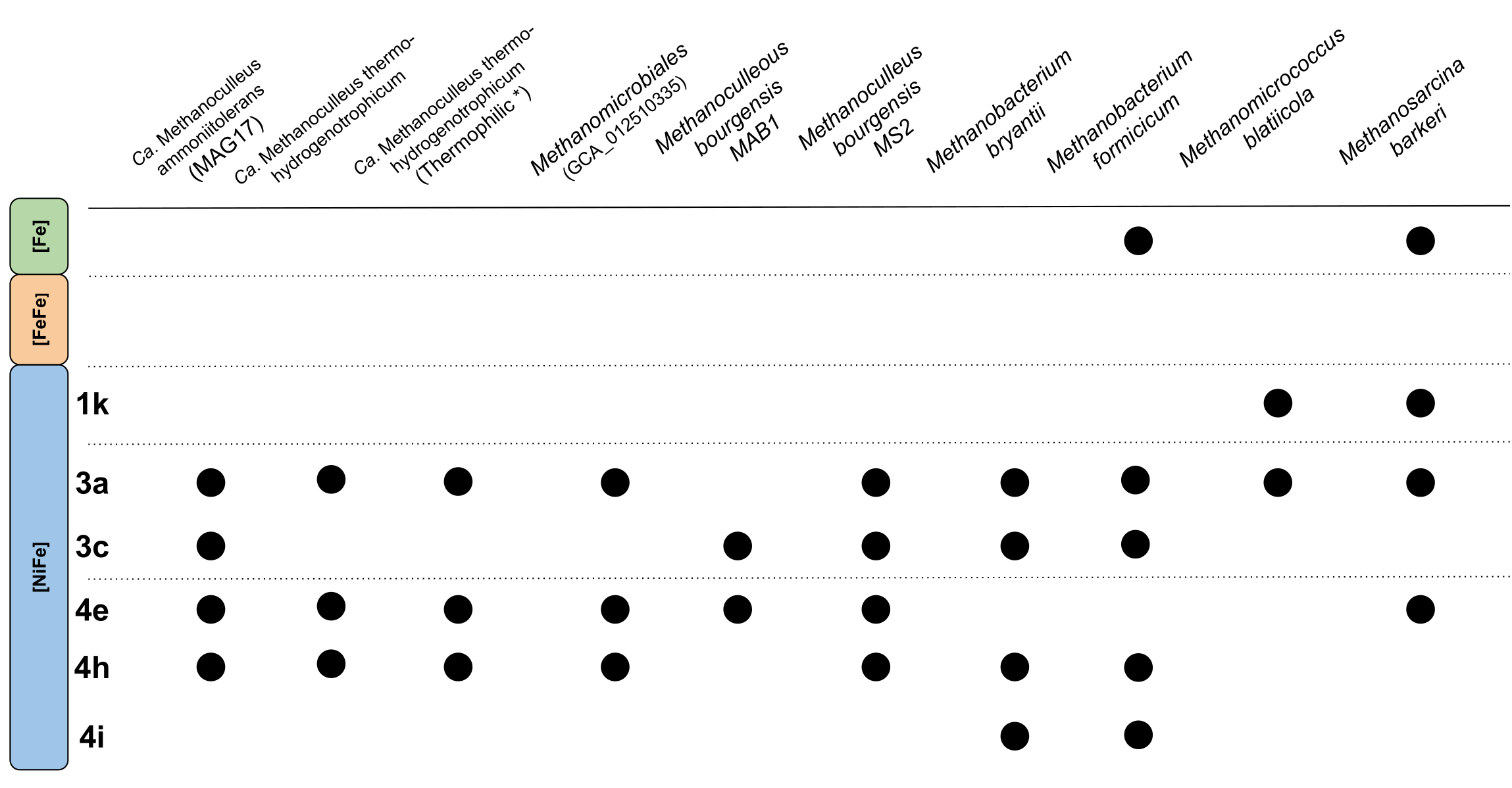


**Fig. S11** Presence of hydrogenases in ‘*Ca.* Methanoculleus ammoniitolerans’ (MAG17) identified in the present study and closely related species and other known hydrogenotrophic methanogens. *Thermophilic ammonia-tolerant hydrogenotrophic methanogen (Singh et al., 2023).


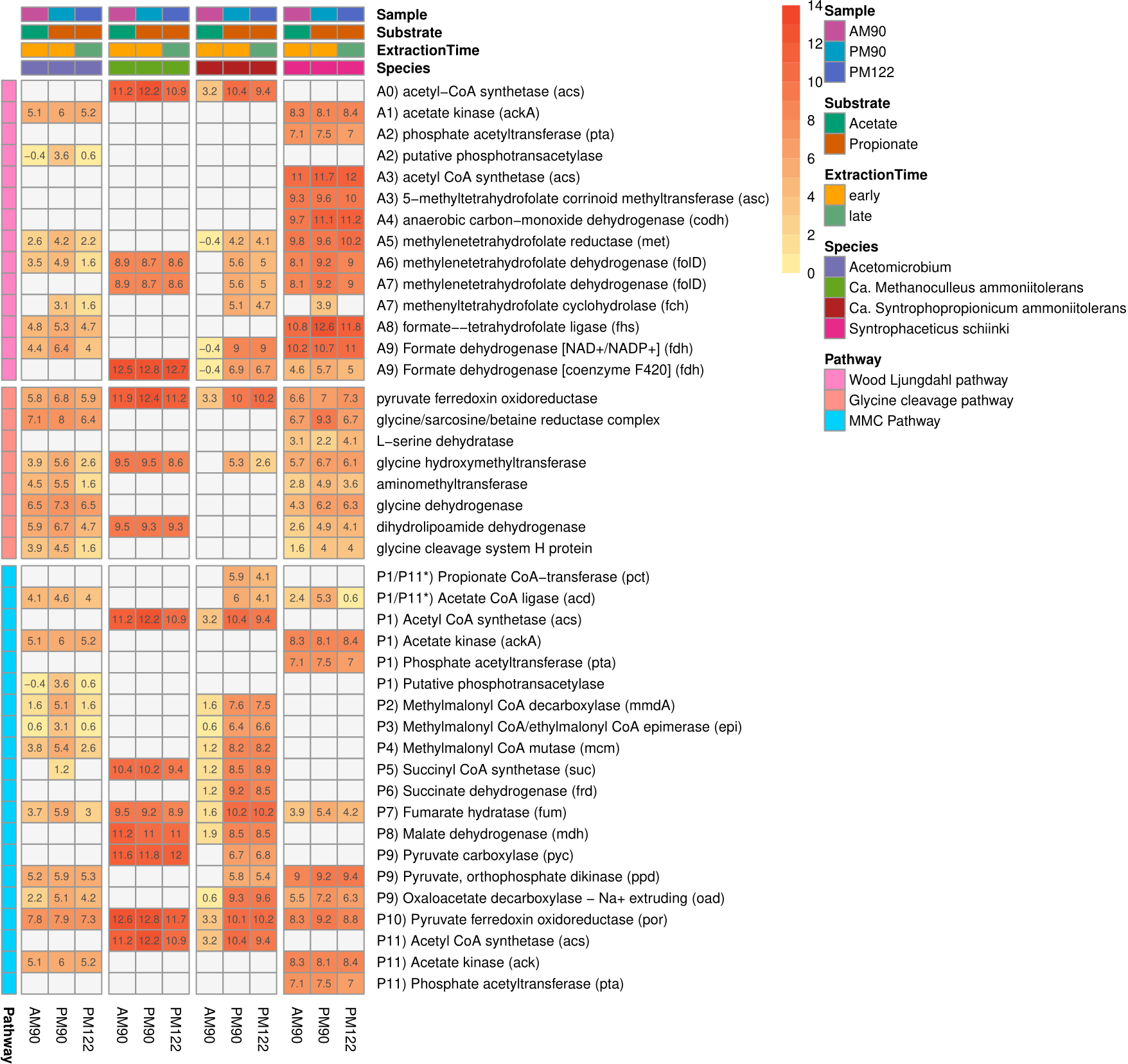


**Fig. S12** Expression of genes encoding enzymes involved in the Wood-Ljungdahl, glycine cleavage and methylmalonyl-CoA pathways by the SAOB *Syntrophaceticus schinkii* (MAG18), the SPOB candidate ‘*Candidatus* Syntrophopropionicum ammoniitolerans’, the methanogen ‘*Candidatus* Methanoculleus ammoniitolerans’ (MAG17) and MAG16 belonging to the genus *Acetomicrobium*. Heatmap values are the aggregated log2-transformed deseq2 normalized count, of all copies and subunits for each respective gene. Genes not expressed are shown in grey. Enzymes involved in putative coupling between step P1 and P11 for propionate activation are marked *.


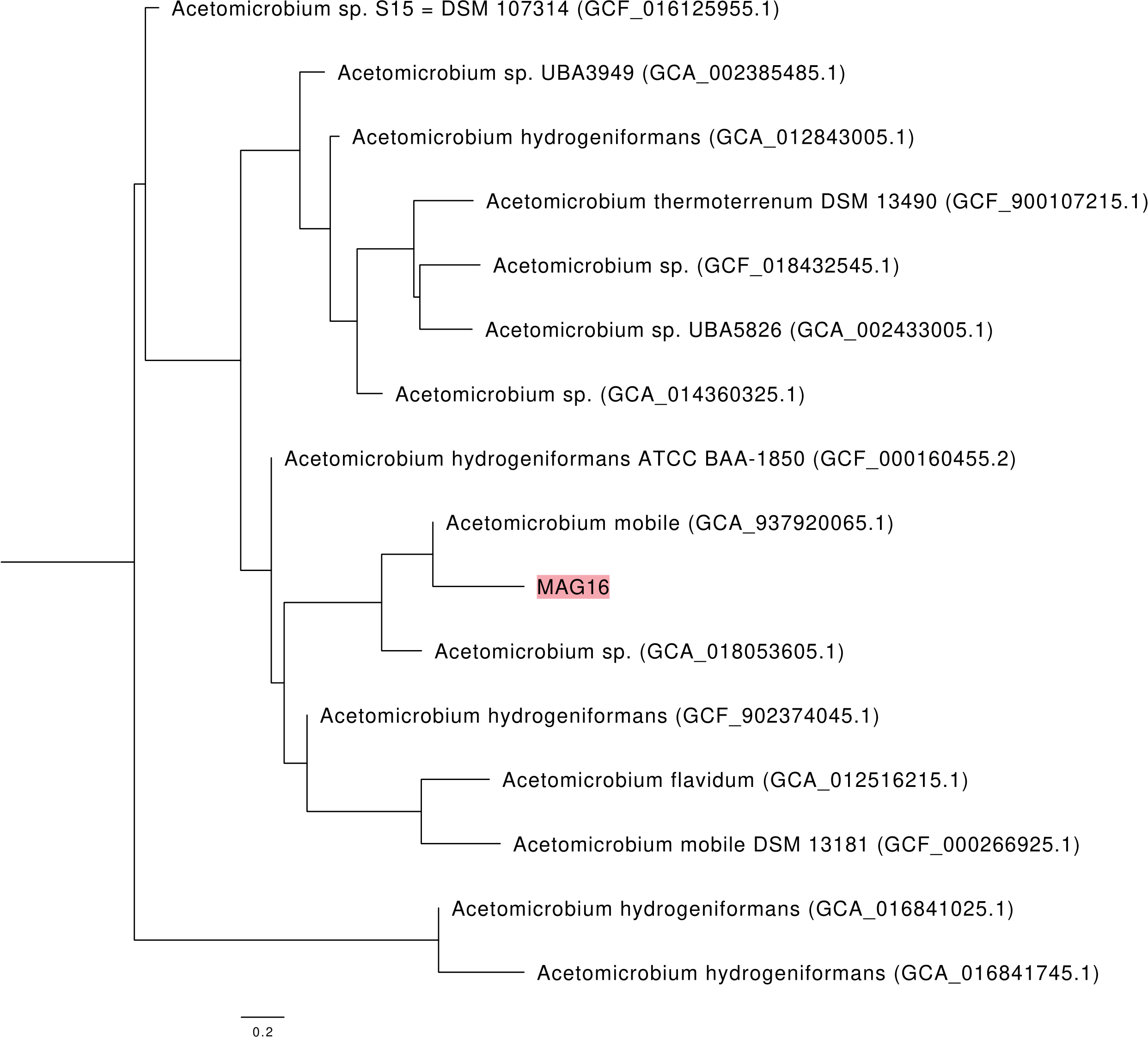


**Fig. S13** Phylogenetic relationships of *Acetomicrobium* (MAG16) with characterized or putative genomes available at NCBI belonging to the genus *Acetomicrobium*.





**Fig. S14** Total ammonia nitrogen (g NH_3_-N/L) in batch assays used for degradation dynamics (aA50, A50, P50, P100). Calculations were based on an initial concentration of 0.3M NH_4_Cl and converted into total ammonia as a function of pH as described by Hansen et al. (1998). This was made with the assumption that all of the supplemented ammonia remained within the ammonia/ammonium form and was not converted to other compounds. Error bars indicate the standard deviation of replicates within each batch assay.

The mean of the triplicates varied between: A50 (0.11 – 0.44 g NH_3_-N/L), aA50 (0.11 – 0.42 g NH_3_-N/L), P100 (0.08 – 0.58 g NH_3_-N/L) and P50 (0.07 – 0.31 g NH_3_-N/L).


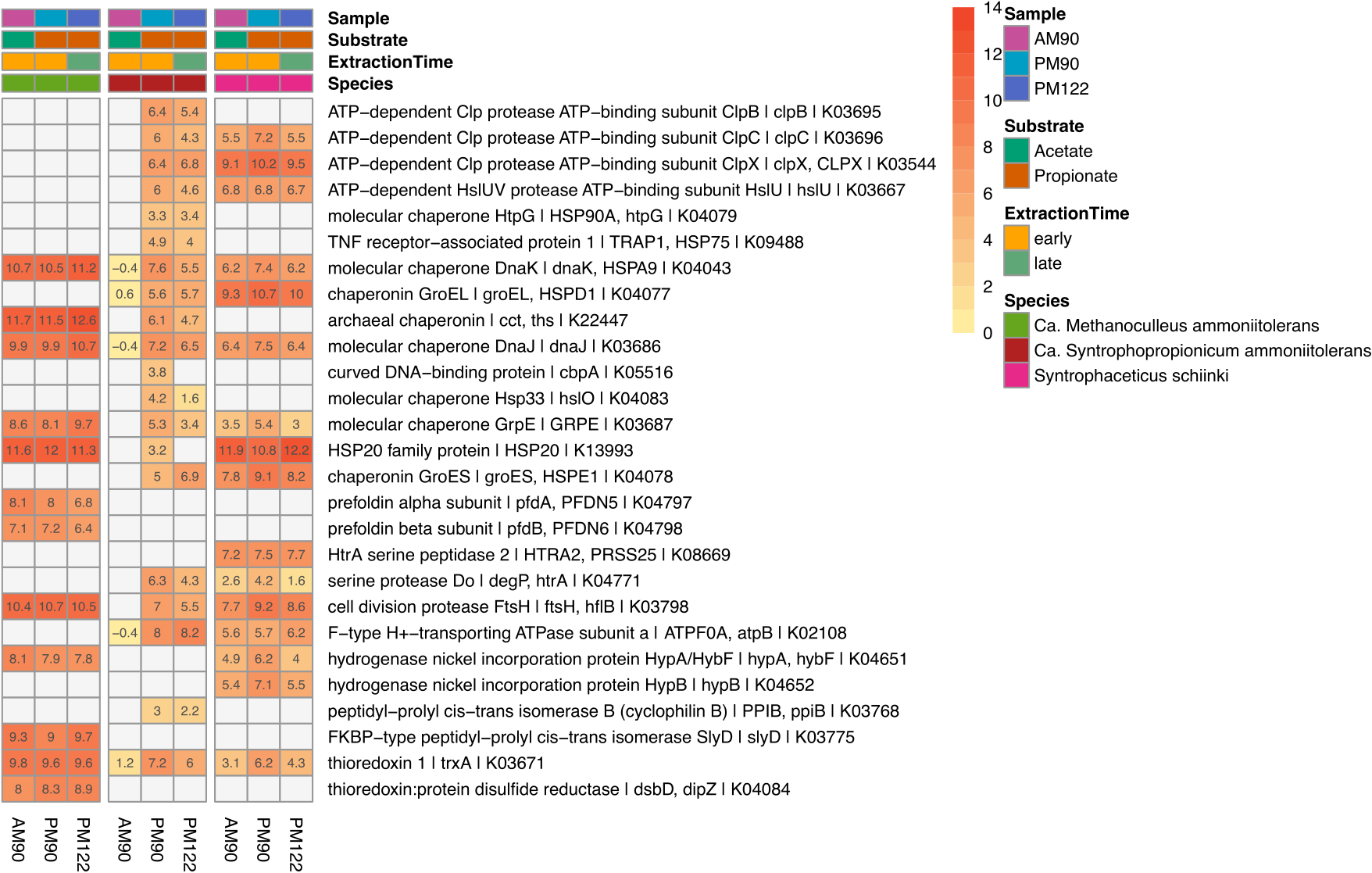


**Fig. S15** Metatranscriptomics expression profile of stress-related proteins of acetate-degrading and propionate-degrading (early/late phase) batches for the SAOB *Syntrophaceticus schinkii* (MAG18), the SPOB candidate ‘*Candidatus* Syntrophopropionicum ammoniitolerans’ (MAG15) and the methanogen ‘*Candidatus* Methanoculleus ammoniitolerans’ (MAG17). Heatmap values are the aggregated log2-transformed deseq2 normalized count, of all copies for each respective gene. Genes not expressed are shown in grey. Row annotations are based on KEGG annotations and are given as follows: Gene name | Protein Symbol | KO identifier

**2 Supplementary Notes**

**Supplementary Note 1**

The putative SPOB *Ca.* S. ammoniitolerans (MAG15)

Following the propionate-activating step in the methylmalonyl–coenzyme A pathway (mmc) and the carboxylation of propionyl-CoA to methylmalonyl-CoA, the methylmalonyl-CoA epimerase and mutase for isomerisation to succinyl-CoA (steps p3-4, Fig. 4, 5) were detected. For the subsequent conversion to succinate (P5) and succinate to fumarate (P6), succinyl-CoA synthetase and the membrane-bound succinate dehydrogenase were expressed. Expression of genes for fumarate hydratase and malate dehydrogenase indicated involvement in conversion of fumarate to malate and then to oxaloacetate. For conversion from pyruvate to acetyl-CoA (p10) pyruvate-ferredoxin oxidoreductase *por* and the gamma subunit were expressed for both the early (PM90) and late sampling point (PM122). Lower expression levels for the alpha, beta and delta subunits were detected in the earlier propionate sample, whereas no expression for these subunits was found in the late-stage propionate sample. Other than the pyruvate-ferredoxin oxidoreductase, coinciding gene expression for the MMC pathway was found in both early and late sampling points.

**Supplementary Note 2**

The SPOB (MAG15) expressed two copies of *fdhC* encoding formate transporters, which resided on the same operon as genes encoding *nuoF* subunits, formate dehydrogenase (fdhA) and two copies of molybdopterin molybdotransferase (*moeA*). The SAOB (MAG18) instead had one gene encoding formate transporter (*fdhC*) with intermediate expression level. This gene resided in an operon containing several hypothetical proteins and surrounding proteins gave blast results similar to FAD dependent oxidoreductases

**3 Supplementary tables**

**Table S1.** Taxonomy of MAG15, MAG16, MAG17 and MAG18 originating from syntrophic propionate-oxidizing enrichment culture. The closest related species were based on closest neighbor in species trees implemented in orthofinder (v 2.5.4) (data not shown here)

|  | **MAG18 (SAOB)** | **MAG15 (SPOB)** | **MAG17 (methanogen)** | **MAG16 (*Acetomicrobium*)** |
| --- | --- | --- | --- | --- |
| Phylum | Firmicutes_B | Firmicutes_B | Halobacteriota | Synergistota |
| Class | Moorellia | Desulfotomaculia | Methanomicrobia | Synergistia |
| Order | Thermacetogeniales | Desulfotomaculales | Methanomicrobiales | Synergistales |
| Family | Thermacetogeniaceae | Pelotomaculaceae | Methanoculleaceae | Acetomicrobiaceae |
| Genus | *Syntrophaceticus* | *Syntrophopropionicum* | *Methanoculleus* | *Acetomicrobium* |
| Closest species | *Syntrophaceticus schinkii* | *Ca.* Syntrophopropionicum ammoniitolerans | MAG 28^3^ | *Acetomicrobium sp012518015* |
| ANI^1^ | 98.58% | 99.44% | 98.48% | 98.04% |
| DDH^2^ | 87.50% | 95.80% | 85.0% | 81.10% |
| 2nd closest species |  |  | *Ca.* Methanoculleus thermohydrogenotrophicum |  |
| ANI^1^ |  |  | 83.41% |  |
| DDH^2^ |  |  | 27.60% |  |

^1^Average nucleotide identity.

*^2^*DNA-DNA hybridization.

^3^ (Singh et al., 2021)

**Table S2.** General genome properties of the ammonia-tolerant syntrophic propionate-oxidizing candidate ‘*Candidatus* Syntrophopropionicum ammoniitolerans’ MAG17, the syntrophic acetate oxidizing bacterium *Syntrophaceticus schinkii* MAG15 and the novel ‘*Candidatus* Methanoculleus ammoniitolerans’ MAG17

| **MAG affiliation** | **MAG** | **Size (Mbp)** | **Number of contigs** | **GC content** | **CDS** | **gene** | **rRNA** | **tRNA** | **Completeness** | **Contamination** | **Strain heterogenity** |
| --- | --- | --- | --- | --- | --- | --- | --- | --- | --- | --- | --- |
| *S. schinkii* | 18 | 2.72 | 8 | 47.26% | 3135 | 3193 | 6 | 48 | 97.41 | 5.17 | 100.00 |
| *Ca*. S. ammoniitolerans | 15 | 2.66 | 2 | 46.76% | 4418 | 4484 | 9 | 48 | 82.13 | 1.50 | 0.00 |
| *Ca*. Methanoculleus ammoniitolerans | 17 | 2.68 | 5 | 58.35% | 3344 | 3396 | 3 | 48 | 90.72 | 2.61 | 100.00 |
| *Acetomicrobium* | 16 | 2.28 | 10 | 44.94% | 2437 | 2502 | 6 | 53 | 100.0 | 0.00 | 0.00 |

^1^Average nucleotide identity

**References**

Hansen, K. H., Angelidaki, I., and Ahring, B. K. (1998). ANAEROBIC DIGESTION OF SWINE MANURE: INHIBITION BY AMMONIA. *Water Research* 32, 5–12. doi: 10.1016/S0043-1354(97)00201-7

Keller, A., Schink, B., and Müller, N. (2019). Energy-Conserving Enzyme Systems Active During Syntrophic Acetate Oxidation in the Thermophilic Bacterium *Thermacetogenium phaeum*. *Front Microbiol* 10, 2785. doi: 10.3389/fmicb.2019.02785

Singh, A., Schnürer, A., Dolfing, J., and Westerholm, M. (2023). Syntrophic entanglements for propionate and acetate oxidation under thermophilic and high-ammonia conditions. *ISME J*, 1–13. doi: 10.1038/s41396-023-01504-y

Singh, A., Schnürer, A., and Westerholm, M. (2021). Enrichment and description of novel bacteria performing syntrophic propionate oxidation at high ammonia level. *Environmental Microbiology* 23, 1620–1637. doi: 10.1111/1462-2920.15388
